# Identification of tomato accessions as source of new genes for improving heat tolerance: from controlled experiments to field

**DOI:** 10.1186/s12870-021-03104-4

**Published:** 2021-07-22

**Authors:** María José Gonzalo, Inmaculada Nájera, Carlos Baixauli, David Gil, Teresa Montoro, Vicky Soriano, Fabrizio Olivieri, Maria Manuela Rigano, Daniela Ganeva, Stanislava Grozeva-Tileva, Galina Pevicharova, Amalia Barone, Antonio Granell, Antonio José Monforte

**Affiliations:** 1 Instituto de Biología Molecular Y Celular de Plantas, Universitat Politècnica de València-Consejo Superior de Investigaciones Científicas, Ingeniero Fausto Elio s/n, 46022 Valencia, Spain; 2 Centro de Experiencias Cajamar Paiporta, Valencia, Spain; 3 Enza Zaden Centro de Investigación S.L, Almería, Spain; 4 Department of Agricultural Sciences, University of Naples Federico II, Portici, Italy; 5 Maritsa Vegetable Crops Research Institute, Plovdiv, Bulgaria

**Keywords:** Climate change, Germplasm, Abiotic stress, Fruit set

## Abstract

**Background:**

Due to global warming, the search for new sources for heat tolerance and the identification of genes involved in this process has become an important challenge as of today. The main objective of the current research was to verify whether the heat tolerance determined in controlled greenhouse experiments could be a good predictor of the agronomic performance in field cultivation under climatic high temperature stress.

**Results:**

Tomato accessions were grown in greenhouse under three temperature regimes: control (T1), moderate (T2) and extreme heat stress (T3). Reproductive traits (flower and fruit number and fruit set) were used to define heat tolerance. In a first screening, heat tolerance was evaluated in 219 tomato accessions. A total of 51 accessions were identified as being potentially heat tolerant. Among those, 28 accessions, together with 10 accessions from Italy (7) and Bulgaria (3), selected for their heat tolerance in the field in parallel experiments, were re-evaluated at three temperature treatments. Sixteen tomato accessions showed a significant heat tolerance at T3, including five wild species, two traditional cultivars and four commercial varieties, one accession from Bulgaria and four from Italy. The 15 most promising accessions for heat tolerance were assayed in field trials in Italy and Bulgaria, confirming the good performance of most of them at high temperatures.

Finally, a differential gene expression analysis in pre-anthesis (ovary) and post-anthesis (developing fruit) under heat stress among pairs of contrasting genotypes (tolerant and sensitive from traditional and modern groups) showed that the major differential responses were produced in post-anthesis fruit. The response of the sensitive genotypes included the induction of HSP genes, whereas the tolerant genotype response included the induction of genes involved in the regulation of hormones or enzymes such as abscisic acid and transferases.

**Conclusions:**

The high temperature tolerance of fifteen tomato accessions observed in controlled greenhouse experiments were confirmed in agronomic field experiments providing new sources of heat tolerance that could be incorporated into breeding programs.

A DEG analysis showed the complex response of tomato to heat and deciphered the different mechanisms activated in sensitive and tolerant tomato accessions under heat stress.

**Supplementary Information:**

The online version contains supplementary material available at 10.1186/s12870-021-03104-4.

## Background

Under the current global warming experienced, the increase in temperatures is expected to be between 2 and 5 °C by the end of the twenty-first century. These temperatures will affect tropical and subtropical temperate regions and will cause losses in agronomic yield proportional to their increase [1, 2]. The adaptation of seed varieties and management practices to warmer temperatures are needed to maintain crop productivity [3]^.^ Also, extreme weather events will be more frequent, contributing more serious threats to crop productivity [4]. The negative effect of the increase in temperature on plant growth and yield has already been reported for many crops such as wheat, rice, barley, sorghum, maize, chickpea, canola [5–7] and tomato, with yield losses of up to 28% [7]. The high temperature stress is determined by two major factors: duration and intensity [8]. Four major thermotolerance responses have been described [9]: short-term acquired thermotolerance, long-term acquired thermotolerance, basal thermotolerance, and thermotolerance to moderately high temperatures.

Tomato (*Solanum lycopersicum* L.) is one of the most important horticultural crops worldwide. Moreover, tomato is cultivated in different climate regions, and it is often exposed to high temperature stress either in greenhouses or when cultivated in fields. The optimal temperatures for tomato growth are between 25°C and 30°C during the day and 20°C at night [10]. However, the increase in these values by a few degrees, such as over 35°C during the day and/or 30°C at night, decreases fruit set due to different factors such as the pollen viability, vegetative growth restraint, drop in flower number or ability of the flowers to set fruits, with the corresponding reduction in yields [11]. A decrease in flower number due to heat stress has also been observed [12–14]. The most commonly-used criterion for defining tomato tolerance is the ability of plants to set fruit after the exposure to high temperatures. This trait is quite complex, involving physiological, biochemical, and gene regulation pathways [15], ranging from pollen viability [16–18], maintenance of photosynthesis and respiration [19], to activation or silencing of genes [20, 21]. Also, fruit set is directly correlated with the final agronomic yield. Therefore, fruit set at high temperature is considered a good indicator of heat tolerance in tomato and has been widely studied in previous works [13, 14, 18, 22].

The exploration of natural variation may offer insights into the genetics of stress tolerance, and can provide genetic diversity that is useful for breeding [23]. Large scale genomic resources can be used to obtain insights into the control of complex abiotic stresses such as heat stress [7]. However, the screening of tomato collections for heat tolerance has only been performed using small collections of accessions [22]. Nevertheless, wild relatives of tomato have been exploited as sources of tolerance to abiotic stresses and diseases. Thus, accessions from wild *Solanum* spp. such as *S. pimpinellifolium*, L., *S. pennellii* L., *S. habrochaites* L., *S. chmielewskii* L. and *S. cheesmaniae* L. have been found to be tolerant to high temperatures [17, 18, 24–27]. Moreover, most of the experiments were conducted under controlled conditions in greenhouses. Physiological measurements, such as chlorophyll fluorescence, have been proposed as quick tools for screening heat tolerance in tomato [28–30]. In some cases, interesting and good correlations have been found between chlorophyll fluorescence measurements in controlled greenhouse and field performance studies under high temperatures [28, 29], although these correlations have not been found with crop yield in all the studies [28]. However, the number of tested accessions and trials is still too low to draw general conclusions. Therefore, even though the screening of tomato germplasm under controlled conditions may be useful for identifying candidate tolerant accessions, their response in the field may be different, as the heat stress can be highly variable, and other factors (soil, weather, diseases), which cannot be mimicked in the greenhouse, and which may also affect plant performance, could be found. Thus, the combined analysis in both greenhouse and field provides a more realistic approach for fully investigating the response of tomatoes to high temperatures.

Moreover, to address global warming, one approach that has gathered importance recently is the analysis of the phenotypic plasticity, described as the capacity of accessions to express different phenotypes according to the environmental conditions [31]. Breeders can exploit plasticity by selecting cultivars based on their yields in different environments to maximize productivity [32]. This approach has been used for studying the tolerance to biotic and abiotic stresses of different crops such as maize [32], sunflower [33], or tomato [34]. The analysis of multiple trials is necessary for precisely estimating the plasticity capacity of the genotypes.

Advances in the knowledge on the mechanisms underlying the tolerance to high temperature will help with developing new thermotolerant cultivars. The use of RNA sequencing (RNA-Seq) technology, a powerful tool for gene discovery, has been used for differentially expressed gene (DEG) analysis of plants grown under different abiotic stresses [35], revealing some candidate genes responsible for the high temperature response [36].

The capacity of genotypes to adapt to stressful conditions entails the activation of different response mechanisms. The reaction of plants to heat stress has been described as a complex trait that affects processes at the morphological, physiological, or molecular levels [8, 25]. Thus, the response to heat stress at the transcriptomic level will be conditioned by the process or processes affected. In plants, heat stress induces the expression of heat shock proteins (HSPs) [37], stress-related proteins and protection against reactive oxygen species [8], and plant hormones and reactive oxygen species [38]. HSPs prevent cellular damage through their activity as chaperons, whereas under non-stress conditions, their role is to assist in the synthesis and transport of other proteins [39]. Even though the first response to heat stress is the activation of HSPs, the role of other genes involved in various biosynthetic pathways, transduction of secondary metabolites, or osmoprotectants (proline, glycine betaine, polyamines, ABA) has also been described in the thermotolerant response of plants [5, 8, 40]. In tomato, several genes involved in heat tolerance have been described [41–43], although due to the complexity of this stress, the knowledge about the mechanisms underlying the tolerance has not yet been fully deciphered.

The main objective of the current research is to test whether heat tolerance determined by a specific physiological trait (capacity to set fruit under high temperature) in controlled greenhouse experiments can be a good predictor of the agronomic performance in field cultivation under high climatic temperature stress.

Heat tolerance was screened in the largest germplasm collection explored until now. The germplasm consisted of tomato accessions or varieties from a very broad origin: wild species, early domesticates, traditional and modern varieties. The heat tolerance response was determined by evaluating reproductive traits such as the number of flowers and fruits produced per truss and fruit set percentage. The accessions were analyzed under different controlled temperature conditions and in natural field conditions in the hottest seasons to verify the stability of the heat tolerance response. Moreover, an RNA-Seq analysis was performed to identify genes involved in the mechanisms used by tomatoes from different groups to overcome the stress due to high temperatures, by using two contrasting genotypes from modern cultivars and the “de penjar”/”da serbo” groups to unravel the mechanisms involved in heat stress tolerance.

## Results

**Fig. 1:**
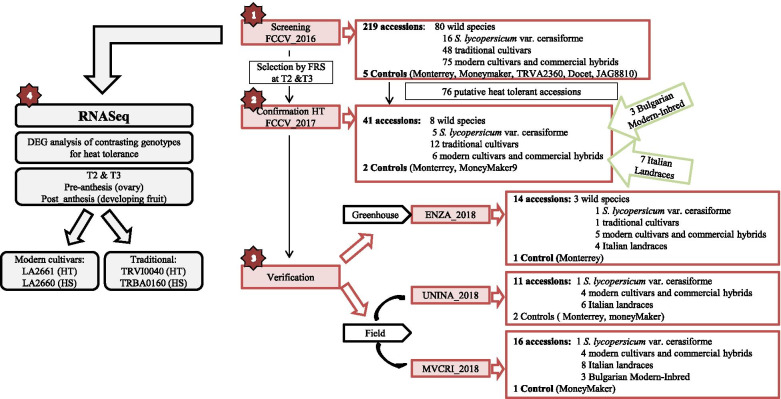
Scheme of the experiments included in the current work: 1) Screening FCCV_2016. Large-scale screening for heat tolerance in tomato germplasm carried out in FCCV in 2016 at three temperature regimes (T1: 25 °C day/ 20 °C night; T2: 30 °C day/ 25 °C night; T3: 35 °C day/ 30 °C night. 2) Confirmation HT FCCV 2017. Confirmation in greenhouse at three temperature regimes (T1: 25 °C day/ 20 °C night; T2: 30 °C day/ 25 °C night; T3: 35 °C day/ 30 °C night) the heat tolerance for 41accessions selected in the previous screening experiment.. 3) Verification of heat tolerance of some tomato accessions: 3.1) under greenhouse conditions, including the ENZA-2018 experiment and 3.2) in the field, with experiments in two open fields in Bulgaria (A) Experiment MCVRI_2018 and (B) in Italy UNINA_2018. 4) RNA-Seq analysis of genotypes with contrasting heat tolerance

Several interrelated experiments were carried out to identify heat tolerant tomato cultivars and accessions as well as to provide some insights on the molecular tolerance mechanisms. A scheme of those experiments and their relationships is shown in Fig. 1.

### Large-scale screening for heat tolerance in tomato germplasm

Our primary objective was to find sources of heat tolerance in tomato for their introduction into breeding programs. We screened the putative heat tolerance of a large number of tomato accessions belonging to diverse groups (wild accessions, *S. lycopersicum* var. *cerasiforme*, traditional cultivars including “de penjar or “da serbo” tomatoes, modern cultivars and commercial hybrids) to cover the greatest number of tomato types from the available germplasm of this species (Fig. 1). Also, five tomato varieties were used as a control.

The response to heat stress of 219 tomato accessions was studied in the FCCV_16 experiment (Supplementary Table 1) with five tomato varieties as controls (‘MoneyMaker’, TRVA2360, ‘Docet’, ‘Monterrey’ and ‘JAG8810’). The screening was carried out under three different temperature regimes T1 (25 °C/20 °C, no heat stress), T2 (30 °C/25 °C, moderate heat stress) and T3 (35 °C/30 °C, extreme heat stress). The use of and augmented design implied the adjustment of the values based on the five results from the controls (Supplementary Table 2).

**Fig. 2:**
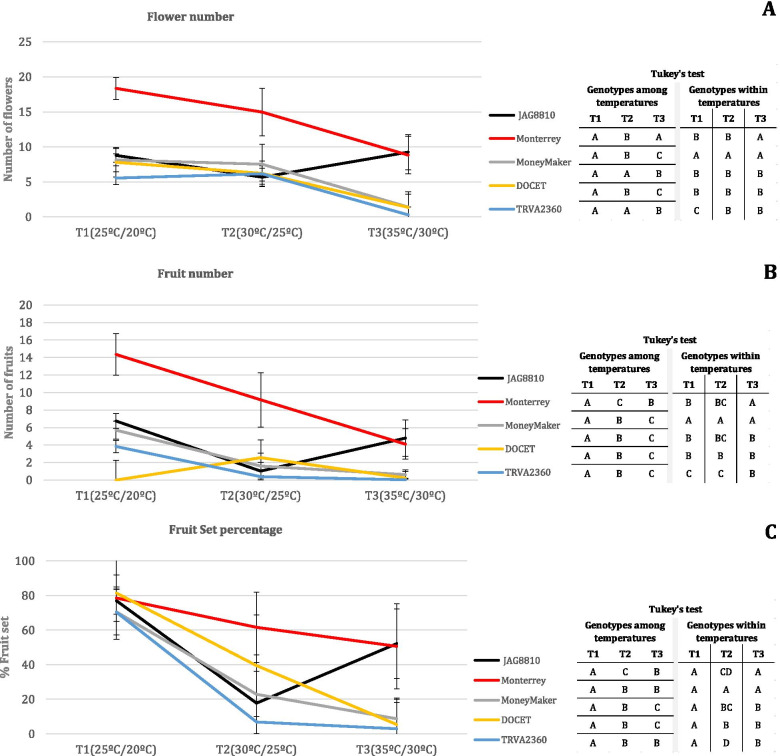
The five control (‘JAG8810’, ‘Monterrey’, ‘MoneyMaker’, ‘Docet’ and TRVA2360) response curves for the traits **(A)** Flower number, **(B)** Fruit number and **(C)** Fruit Set percentage, in the three temperature regimes (T1: 25 °C/20 °C, T2: 30 °C/25 °C and T3: 35 °C/30 °C) in FCCV_2016 experiment are depicted. The comparison of the each mean genotype among temperatures and among genotypes within each temperature regime was carried out by a Tukey test (*p *< 0.05) and the results are depicted in the tables on the right side of the figure. The same letter indicates equal mean

Figure 2 shows the trend of the responses of the controls to the increase in temperature. A two-way ANOVA showed that genotype, temperature effects and their interaction were highly significant (*p *< 0.001) for all three traits. In general, the response of genotypes among the range of temperatures was a reduction in FLN (flower number) (Fig. 2A), FRN (fruit number) (Fig. 2B) and FRS (Fruit Set percentage) (Fig. 2C) concomitant with the temperature increase, except for ‘JAG8810”. ‘Monterrey’ showed a significantly higher FLN, FRN and FRS at T2 and T3 than “Moneymaker”, “DOCET” and TRVA2360, demonstrating its heat tolerance. Regarding ‘JAG8810’, a severe FLN, FRN and FRS decrease was observed at T2 (similar to “MoneyMaker” and TRVA2360), although all of them increased in T3, with the behavior at this temperature regime being similar to ‘Monterrey’. Therefore, three patterns were found (Fig. 2): a sensitive pattern (drastic reduction observed in ‘MoneyMaker’, ‘DOCET’ and TRVA2360), a tolerant pattern (mild reduction observed in “Monterrey”) and an adaptive pattern (reduction initially but recovery later in “JAG8810″).

**Fig. 3:**
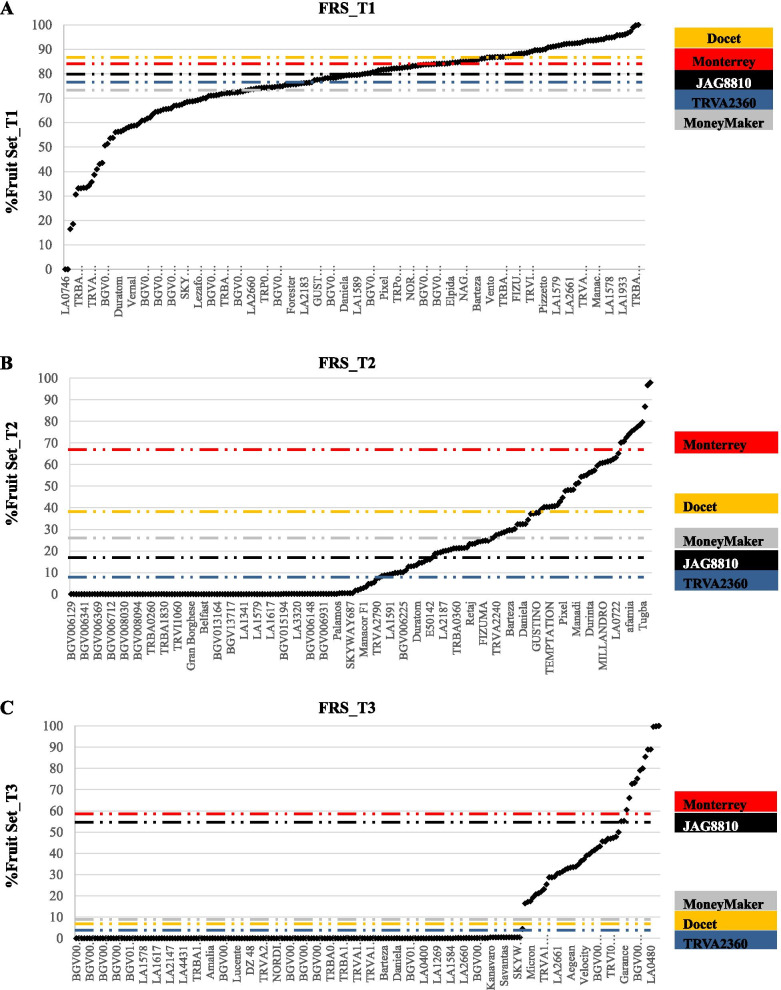
Percentage of Fruit Set (FRS) in T1 **(A)**, T2 **(B)** and T3**(C)** for the 219 accessions in the FCCV_2016 experiment. Accessions are depicted in the X axis, although all the names did not fit in the figure, so a sample of them is mentioned. FRS for each accession is indicated with a black dot. Dashed colored horizontal lines correspond with the statistical threshold (*p *< 0.05) to declare significant differences between accessions and controls: yellow (‘DOCET’), blue (TRVA2360), grey (‘MoneyMaker’), black (‘JAG8810’) and red (‘Monterrey’)

The 219 tested accessions also showed differences in FLN, FRN, and FRS among the three temperature regimes. Regarding FLN, the differences observed between accessions at T1 and T2 were small, as confirmed by the high correlation (r = 0.74, Supplementary Fig. 1) observed for this trait between these two temperature regimes. Indeed, only a slight decrease in FLN was observed at the higher temperature T2. However, FLN decreased drastically in most of the accessions at T3 (Supplementary Fig. 1). In the case of FRN, a high decrease was already observed at T2, where 63% of the accessions did not produce fruits (Supplementary Fig. 1). Following this downward trend, only 22% of the accessions set fruits at T3 (Supplementary Fig. 1). In general, most of the accessions showed a high FRS at T1, whereas different responses were observed at T2 and T3. Of all the accessions, 37% and 22% of them did not set fruit at T2 and T3, respectively, whereas 5% showed a high FRS (> 70%) at both high temperature treatments (Fig. 3). Therefore, 51 and 47 accessions had statistically significant higher FRS than the sensitive controls at T2 and T3, and 22 of them at both temperature regimes (Fig. 3, Supplementary Table 3). Remarkably, 12 and 13 accessions showed significantly higher FRS than the tolerant controls ‘Monterrey’ and ‘JAG8810’, at T2 and T3, respectively (Fig. 3C).

To homogenize the analysis for the sex set of experiments, ‘MoneyMaker’ and ‘Monterrey’ were defined as the sensitive and tolerant controls, respectively. Seventy-six accessions (35%) showed a higher FRS than ‘MoneyMaker’ either at T2 or T3. These accessions included 12 wild accessions (15% of the total wild accessions analyzed), four *S. lycopersicum* var. *cerasiforme* accessions (25%), 12 traditional “de penjar” or “da serbo” cultivars (33%), two other traditional cultivars (17%), eight modern cultivars (42%) and 38 commercial hybrids (68%) (Table 1).

**Table 1:** Number and percentage (between brackets) of accessions showing significantly higher tolerance to high temperatures (T2 and T3) than the ‘MoneyMaker’ control for each tomato group in the FCCV_2016 experiment

		Wild	Cerasiforme	Traditional		Modern		**TOTAL**
				“de penjar/da serbo”	traditional	Cultivars	Commercial	
**ANALYZED**		80	16	36	12	19	56	**219**
SELECTED	T2	7	1	1	-	3	17	29
	T2&T3	3	-	2	1	1	15	22
	T3	2	3	9	1	4	6	25
	Total selected	12 (15%)	4 (25%)	12 (33%)	2 (17%)	8 (42%)	38 (68%)	76 (35%)

**Fig. 4:**
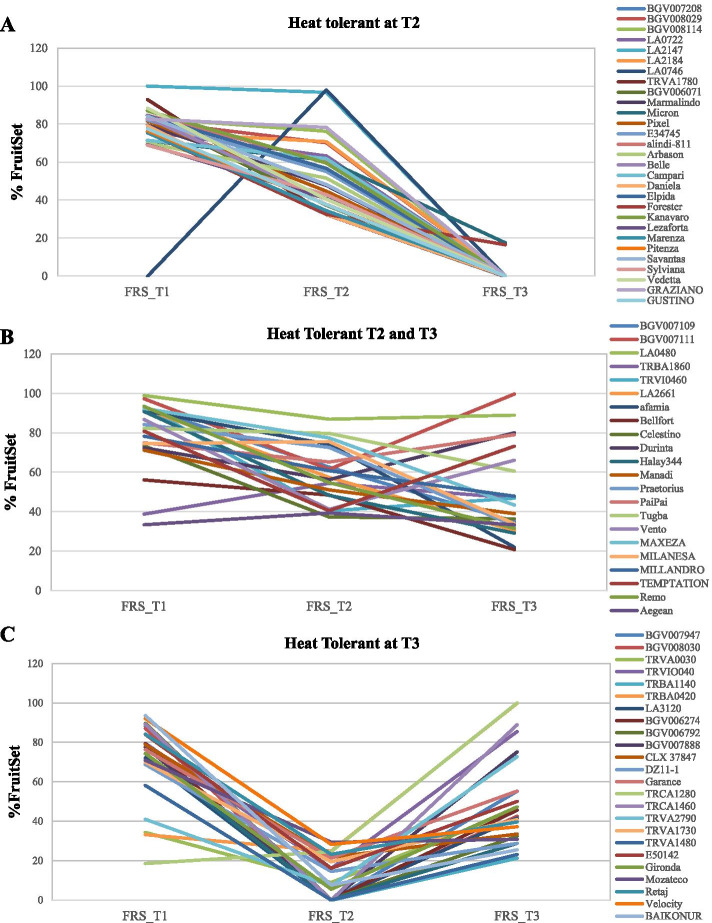
Different Fruit Set percentage (FRS) response trends among temperature regimes (T1, 25 °C/ 20 °C day/night; T2, 30 °C/ 25 °C; T3, 35 °C/30 °C) for the 76 putative heat tolerant genotypes in the FCCV_2016 experiment: **(A)** Heat tolerant in T2: fruit set in T2 higher than sensitive control ‘MoneyMaker’**, (B)** Heat tolerant in T2 and T3: the fruit set in T2 and T3 higher than ‘MoneyMaker’ **(C)** Heat tolerant in T3: fruit set in T3 higher than ‘MoneyMaker’. The statistical threshold to declare significant differences from ‘MoneyMaker’ (*p *< 0.05) was calculated based on the adjusted values from the augmented design

The 76 accessions with a significantly higher FRS than ‘MoneyMaker’ in at least one high temperature regime were considered as high temperature (HT) tolerant candidates, as they showed different response trends at moderate and extreme temperature regimes, T2 and T3 (Fig. 4), as previously observed in the control group. These accessions followed three different behaviors under high temperatures, and could therefore be categorized into three different groups. Most of the accessions with a high FRS at T2 showed a nearly linear FRS decrease with the temperature increase (Fig. 4A), although the FRS reduction was lower than in ‘MoneyMaker’, i. e., these were classified as tolerant to moderate heat stress. Twenty-two accessions showed significantly higher FRS than ‘MoneyMaker’ in both T2 and T3 (Fig. 4B), but two different patterns were observed: fifteen accessions showed a decrease in both T2 and T3, although significantly less than ‘MoneyMaker’, and seven accessions showed a decrease of FRS in T2, followed by an increase in T3. (Fig. 4B). This group was classified as tolerant candidate to a broad range of temperature stresses. Finally, the rest of the tolerant accessions (25) shoed a severe reduction in FRS in T2, but a high FRS (recovery) at T3 (Fig. 4C), following the same pattern already observed in the performance of the tolerant control ‘JAG8810’, and were classified as candidates that could be able to adequately respond to heat stress.

### Confirmation of the putative heat tolerance of tomato accessions in a greenhouse under controlled growing conditions

To confirm the heat tolerance of the genotypes selected from the previous large screening, a trial with five biological replications for each genotype and the same heat treatments was carried out (Fig. 1, FCCV_2017). A total of 41 accessions were analyzed under heat conditions, 31 from the candidate 76 tolerant genotypes selected in the FCCV_2016 experiment (8 wild accessions, 5 *S. lycopersicum* var. *cerasiforme*, 12 traditional cultivars including 7 “de penjar or “da serbo” tomatoes, 6 modern cultivars and commercial hybrids), seven heat tolerant Italian landraces [44, 45], belonging to a collection available at the University of Naples Federico II, Department of Agricultural Sciences which showed heat tolerance in parallel field experiments (details hosted at LabArchive repository http://dx.doi.org/10.6070/H4TT4NXN) and three heat tolerant Bulgarian modern inbreds from MVCRI (details will be published elsewhere). The inclusion of the two later groups was done as part of the coordination efforts within the TOMGEM project.

The FLN values were maintained as in T2, with only a slight decrease observed when compared with the T1 results. However, in the extreme T3 regime, an evident decrease in FLN was observed. For FRN, this decrease occurred in T2, with an extreme drop in the number of fruits in T3. As for FRS, high values were observed in T1 for all the accessions, with a percentage of fruit set varying between 50 and 100%. In T2, a high variability in FRS was observed, ranging from genotypes with no fruit set to genotypes with a 100% FRS. However, in T3 the FRS decreased for almost all the genotypes (Supplementary Fig. 2).

FLN and FRN showed a high correlation in T1 and T2 (0.93 and 0.91, respectively) with the previous FCCV_2016 experiment. However, correlations were very low in T3; r = 0.22 and r = 0.30 for FLN and FRN, respectively. The correlation of FRS among these two experiments was low and significant only in T1 (Table 2).

**Table 2:**
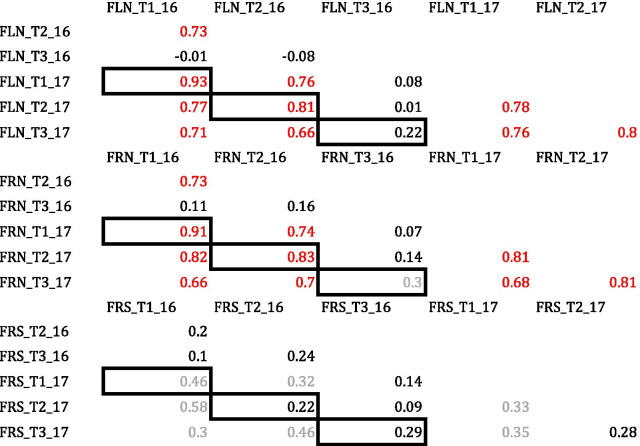
Correlations of FLN (flower number), FRN (fruit number) and FRS (Fruit Set percentage) for experiments FCCV_2016 *vs* FCCV_2017 at the three temperature regimes: T1: 25°C/20°C, T2: 30°C/25°C and T3: 35°C/30°C. Values in red show the correlations that were significant at *p *< 0.01, and values in grey at *p *< 0.05

The wild accessions were analyzed separately from the *S. lycopersicum* species genotypes due to their very different plant phenology. In the case of the wild accessions, FRS was high for all accessions, ranging between 100 and 97% in T1 and without significant differences observed with respect to the controls. In T2, only one wild species (BGV007947) showed a lower FRS than ‘Monterrey’, confirming the heat tolerance of the selected wild accessions. Furthermore, five accessions showed significantly higher FRS than ‘MoneyMaker’. In T3, the differences between accessions were more evident. Three genotypes showed the same response as ‘Monterrey’, and four accessions were significantly better than ‘MoneyMaker’ for FRS (Table 3 A).

**Table 3:**
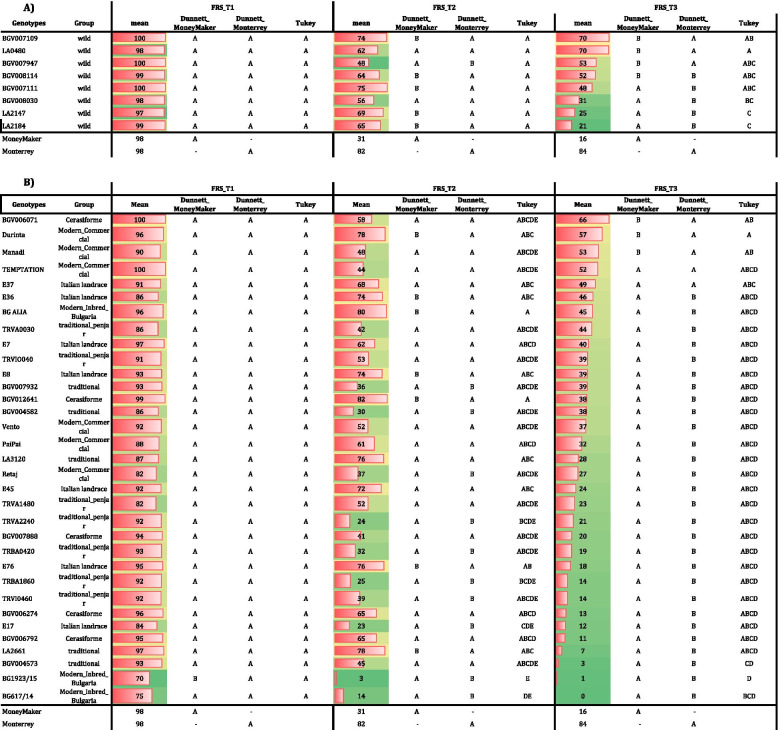
Dunnett’s test comparing the 41 putative heat tolerant genotypes and the controls ‘MoneyMaker’ and ‘Monterrey’, and Tukey’s test for the comparison between accessions in the FCCV_2017 experiment. (**A**) wild species (**B**) *S. lycopersicum*species in the three temperature regimes (T1: 25°C/20°C, T2: 30°C/25°C and T3: 35°C/30°C)

For the *S. lycopersicum* varieties and accessions, the FRS in T1 ranged between 100 and 70%, and generally no differences were found with both controls. In T2, 23 genotypes showed a similar response to the tolerant control ‘Monterrey’: six traditional varieties, five *cerasiforme* types, five modern hybrids, one modern inbred from Bulgaria and six Italian landraces, thereby confirming their tolerance to high temperatures. In T2 temperature regime, seven accessions were significantly better than ‘MoneyMaker’. At the extreme high temperature regime, T3, five accessions showed the same FRS as the tolerant control ‘Monterrey’, while three were significantly higher than the sensitive ‘MoneyMaker’ (Table 3 B).

The 41 genotypes were ranked based on their capability to set fruits in each temperature regime. The FRS of the genotypes were compared between themselves using Tukey’s test. The wild species showed similar values for FRS in T1 and T2, without significant differences between accessions. In T2, the FRS ranged between 75% for BG007111 and the 48% for BGV007947. In T3, the FRS was above 20% for all accessions, with four accessions showing a FRS > 50%. Moreover, in T3 significant differences were observed between the two wild accessions with higher values (BGV007109 and LA0480), and the two accessions with the lowest values (LA2147 and LA2148, Table 3 A). Regarding the 33 *S. lycopersicum* accessions, no differences were observed for FRS in T1 between them. Eighteen genotypes showed FRS values above 50%, and only two of the genotypes showed a FRS < 20% in T2 (Table 3 B). Five groups were defined by Tukey’s test, indicating important differences in the response to the T2 regime between accessions. Moreover, in the extreme temperature regime T3, 21 (65%) genotypes showed a FRS > 20% and four higher than 50% (Table 3 B). Four groups were defined by Tukey’s tests, although most accessions did not show significant differences between them. The significant differences were found among the three accessions with higher FRS values (BGV006071, ‘Durinta’ and ‘Manadi’), and the three accessions with the lowest FRS values (BGV004573, BG1923/15 and BG617/14) (Table 3 B).

Twenty-three genotypes did not show significant differences with the tolerant control in T2, indicating that they would be tolerant to moderate high temperature stress. Moreover, three accessions were significantly better for FRS than ‘MoneyMaker’, and five were similar to ‘Monterrey’ at the extreme temperature conditions of T3, resulting in a total of five accessions that were putatively tolerant to extreme temperatures.

### Verification of the heat tolerance of tomato accessions in a greenhouse under controlled growing conditions

A further verification of heat tolerance was carried out in another greenhouse facility (Fig. 1, ENZA_2018), including modern commercial hybrids, wild accessions, Italian landraces groups, traditional “de penjar”/da serbo” tomatoes and one *S. lycopersicum* var. *cerasiforme* accessions. The temperature regimes were similar to FCCV_2017, but the fruits were maintained until harvesting at the optimal ripening stage (i. e., not pruned after the temperature regime change), following an agronomic management similar to commercial production. FRN values were lower than those recorded in FCCV_2017 for all accessions in all temperature regimes (Supplementary Fig. 3) with a subsequent lower FRS, likely due to the different agronomical management (Table 4). Moreover, only the tolerant ‘Monterrey’ could be used as the control due to unexpected problems with the ‘MoneyMaker’ seed lot. Dunnett´s test showed no significant differences between the tested accessions and ‘Monterrey’ in both T2 and T3 regimes (Table 4). The FRS ranged between 40% for E8 and 7% for BGV006071 in T2, and 27% for ‘Durinta’; no fruit set was obtained from E7 and BGV006071 in T3.

**Table 4:**
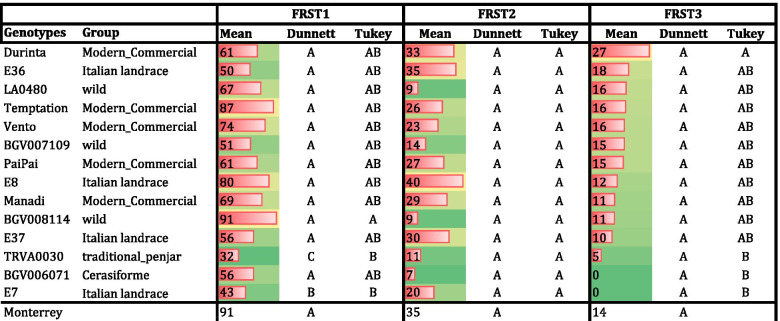
Dunnett’s test comparing FRS (Fruit Set percentage) among the 14 putatively heat tolerant genotypes and the tolerant control ‘Monterrey’, and Tukey’s test for the comparison between accessions in the ENZA_2018 experiment. The wild species and *S. lycopersicum* accessions in the three temperature regimes are reported (T1: 25°C/20°C, T2: 30°C/25°C and T3: 35°C/30°C)

Tukey’s test carried out on 14 accessions revealed similar FRS values for all the accessions T2, and two groups with significant differences in T3, with only ‘Durinta’ (FRS = 27%) being significantly different from BGV006071, E7, and TRVA0030 (Table 4).

Aside from the differences in the agronomic management in the two experiments, the FLN and FRN correlation between the FCCV-2017 and ENZA-2018 experiments was highly significant. The coefficients of correlation obtained for FRS were lower than those from the other two traits but equally significant (Table 5 A).

**Table 5:**
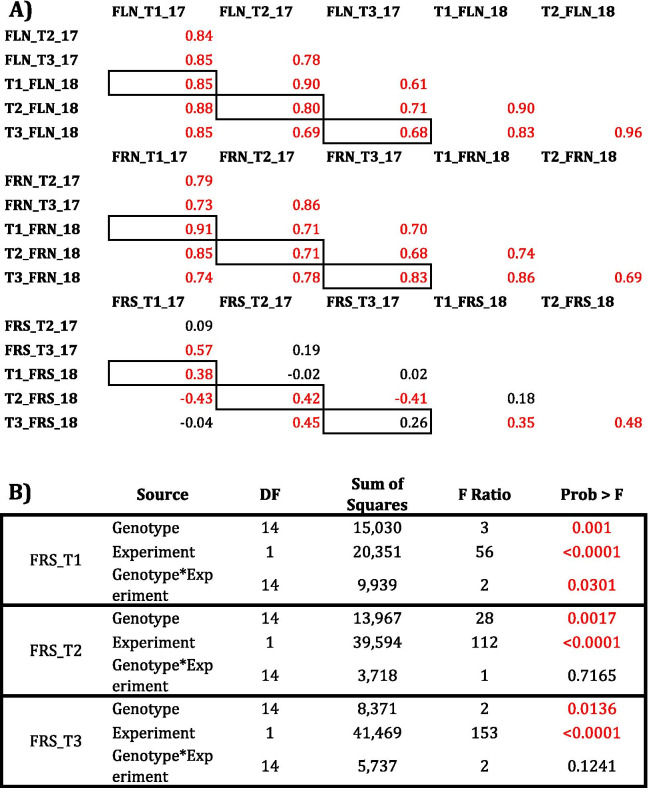
(A) Correlations of FLN (flower number), FRN (fruit number) and FRS (Fruit Set percentage) for the experiments FCCV_2017 *vs* ENZA_2018. Red values show the correlations that were significant at *p *< 0.01. (B) Two-way ANOVA to compare FCCV-2017 and ENZA-2018 experiments for the FRS trait in the three temperature regimes (T1: 25°C/20°C, T2: 30°C/25°C and T3: 35°C/30°C)

Moreover, the analysis of the genotype x environment interaction showed that the differences were due to the genotype and to the experimental conditions but not due to the interaction between factors in the temperature regimes with high temperatures (Table 4 B, supplementary Fig. 4).

Thus, in general, the behavior of the selected accessions and varieties was very similar between them and to the heat tolerant control ‘Monterrey’ reinforcing the hypothesis of their heat tolerance, as observed in the previous experiments.

### Verification of heat tolerance in the field

In order to assess the transferability of the selections based on controlled greenhouse experiments to field grown tomatoes, a group of heat tolerant candidate accessions and cultivars (including modern commercial hybrids and inbreds, Italian landraces and one *S. lycopersicum* var. *cerasiforme* accession) selected from the previous experiments was also tested in two open experimental fields. (Bulgaria, MVCRI_2018, and Italy, UNINA_2018, Fig. 1) with different weather conditions in the warm season.

In the MVCRI_2018 experiment, FLN, FRN and FRS were recorded from four trusses, corresponding to the second to fifth truss of each plant. The mean temperatures from truss appearance to harvest were: truss2, 26 °C day/20 °C night; truss3, 24 °C day/19 °C night; truss4, 26 °C day/20 °C night; truss5, 28 °C day/21 °C night. Therefore, trusses 2, 3 and 4 were not under heat stress whereas truss 5 was under moderate heat stress. In general, no significant differences were found between the tested genotypes and ‘MoneyMaker’ for FLN and FRN. Only BG1923/15 showed higher FLN and FRN than ‘MoneyMaker’, whereas BG617/14 showed FLN and FRN values that were lower than ‘MoneyMaker’ in all the trusses (Supplementary Table 4). Regarding FRS, all the accessions showed a good FRS, in general higher than 50%, with no significant differences with ‘MoneyMaker’ (Table 6 A). The comparison between accessions using Tukey’s test revealed two statistically significant groups in truss4, with the lowest value found for the Italian landrace E17 ( 50% of FRS). These results indicate that the selected heat tolerant varieties did not show yield penalty under absence or moderate heat stress.

**Table 6:**
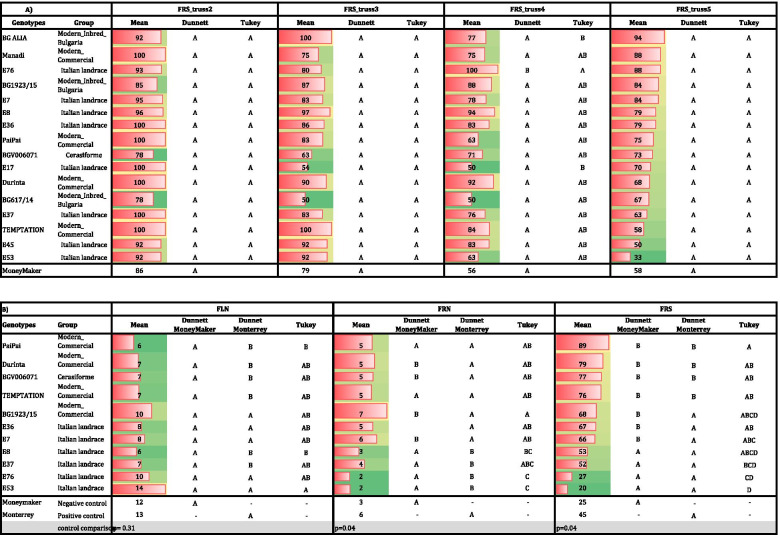
Field experiments: (A) Dunnett’s test for comparing 16 putative heat tolerant genotypes and the control ‘MoneyMaker’, and Tukey`s test for the comparison between accessions in the MVCRI_2018 experiment. (B) Dunnett’s test for comparing 11 putative heat tolerant genotypes and the controls, and Tukey`s test for the comparison among accessions in UNINA_2018 experiment

In the UNINA_2018 field experiment, the maximum temperature even reached 37 °C during the day, and decreased to 17 °C at night, with a mean of 29.7 °C, values which corresponded to a high heat stress during day with a mild temperature during the night, conditions that may help the plant to recover from the diurnal heat stress. In this experiment, the heat sensitive ‘MoneyMaker’ and heat tolerant ‘Monterrey’ were used as negative and positive controls, respectively. No significant differences were found between controls for FLN. However, ‘Monterrey’ did show significantly higher FRN and FRS than ‘MoneyMaker’ (Table 6 B). No differences were also found between ‘MoneyMaker’ and the tested varieties for FLN. On the other hand, five varieties did not show significant differences compared to ‘Monterrey’. In the case of FRN, more significant differences were found: four varieties showed a higher FRN than ‘MoneyMaker’ and seven were not different from ‘Monterrey’. Regarding FRS, seven varieties showed a significant higher FRS than ‘MoneyMaker’, whereas none of them showed a significantly lower FRS than ‘Monterrey’. Remarkably, four varieties showed a higher FRS than ‘Monterrey’ (Table 6 B). In every case, except for two Italian landraces (E53 and E76), the values for FRS were higher than 50% (Table 6 B). Therefore, the behavior of most of the varieties confirmed their heat tolerance in field conditions with respect to the controls. The comparison between accessions with Tukey’s test showed FLN differences for three accessions: E53, which showed the highest flower number, whereas ‘PaiPai’ and E8 showed the lowest ones. Regarding FRN, accession BG1923/15 exhibited the highest value, whereas E76 and E53 obtained the lowest values. Finally, regarding FRS, the lowest values were recorded for E76 and E53, and the highest one for the commercial variety ‘PaiPai’.

### RNA-Seq experiment and analysis

To obtain insights on the molecular mechanisms underlying the heat tolerance, the differential expression of genes among contrasting heat tolerant genotypes was studied with NA analysis. Four genotypes were selected based on the previous results (Fig. 1). Two of them belonged to the traditional group “de penjar/da serbo”, widely cultivated in the Mediterranean area, but with differences in their heat stress response: TRVI0040 (tolerant) and TRBA0160 (sensitive). The similarity in the phenology, fruit type, and vegetative growth of the two accessions made their gene expression pairwise comparison suitable for identifying differential expression due to their different responses to high temperatures, instead of other biological aspects. The other two accessions selected were from the modern cultivar group with different performance under high temperatures: LA2661 (‘Nagcarlang’, tolerant), and LA2660 (sensitive). Accession LA2661was selected because it had been extensively used as a source of heat tolerance in previous studies [12, 18, 46], although in our initial screenings it was only moderately tolerant. TRVI0040 and TRBA0160 showed differences in FLN in T1 and T2 (Table 7 A), even though both varieties decreased FRN in T3 and TRVI0040 had a significantly higher FRN than TRBA0160 in this temperature regime. TRVI0040 showed significant higher FRS in T3, confirming its heat tolerance (Table 7 A).

**Table 7:** FLN (flower number), FRN (fruit number) and FRS (Fruit Set percentage) mean values and statistical comparison (** *p *< 0.01) for comparing the genotypes from (A) traditional (“de penjar/ da serbo group”) TRVI040 (Heat tolerant) vs TRBA0160 (Heat sensitive) and (B) modern cultivars LA2661 (Heat tolerant) vs LA2660 (Heat sensitive)

"de penjar" or "da serbo"		T1		T2		T3	
A) Traditional							
FLN	TRVI0040(HT)	11	**	8.11	**	4.78	
	TRBA0160(HS)	5.11		4.44		3.72	
FRN	TRVI0040(HT)	9.56	**	6.39	**	2.39	**
	TRBA0160(HS)	4.72		3.83		0.56	
FRS	TRVI0040(HT)	87.37		78.71		50	**
	TRBA0160(HS)	92.72		86.34		15.39	
B) Modern							
FLN	LA2661 (HT)	8.06	**	6.94	**	6.36	
	LA2660 (HS)	16		14.56		7.11	
FRN	LA2661 (HT)	7.61	**	6.44		3.17	
	LA2660 (HS)	14.22		9.11		1.22	
FRS	LA2661 (HT)	94.83		92.93	**	49.56	**
	LA2660 (HS)	88.71		62.28		13.34	

Regarding the other two varieties, LA2660 showed a significantly higher FLN than LA2661 in T1 and T2, mainly due to the higher reproductive vigor of this accession. In T3, both accessions showed similar FLN values. However, while LA2661 maintained the number of flowers in the different temperature conditions, a strong decrease of this trait was observed for LA2660. For FRN, the differences in T1 were significant between accessions. However, at high and extreme temperatures, T2 and T3, both accessions showed similar FRN values with a large decrease for the LA2660 accession. Lastly, FRS decreased with increasing temperature in both accessions, although LA2661 showed a higher FRS than LA2660 in all the temperature regimes. In the case of LA2660, the FRS decrease was concomitant with the temperature increase, with a drastic reduction in T3 (Table 7 B). These results confirm the heat tolerance of LA2661 reported in previous works [46] and the lack of it by LA2660.

### Differential gene expression at high different temperatures among genotypes and tissues

**Fig. 5:**
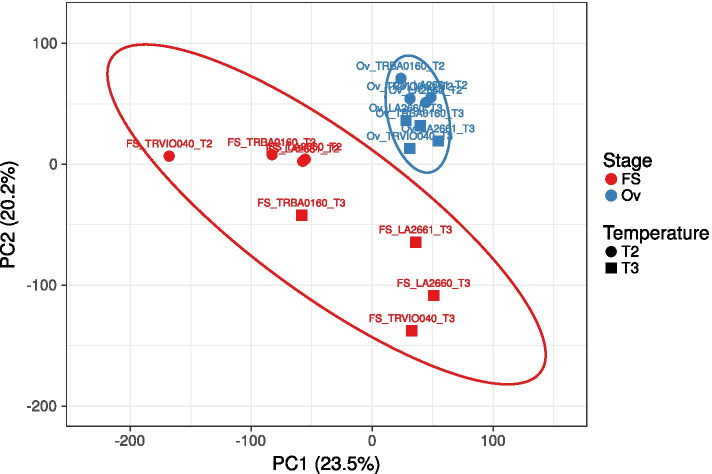
Plot of the two first principal components of PCA of the transcriptome in T2 and T3 for ovaries (Ov) and early developing fruit (FS) stages of two different tomato modern cultivars (LA2661 and LA2660) and “de penjar/da serbo” varieties (TRVI0040 and TRBA0160). Fruit samples are highlighted in red and ovary samples in blue

According our previous results (Gonzalo et al. 2019), the fruit set at high temperatures was dependent on the ovary capacity, not pollen viability as in the current experimental population and experimental set up (we did not exclude that pollen viability could be important in other scenarios). In our experimental set up, the fruit reached only very early development stages before trusses were pruned, which occurred before the change in the temperature regime. We decided to also include early developing fruit (5 days post-anthesis) in the differential expression analysis to expand the scope of the work and to identify candidate genes that could be important in the early development. Therefore, the differential expression at high temperature was studied in two tissues at different developmental stage (pre-anthesis (ovary) and post-anthesis (developing fruit)), and in two different temperature regimes (T2 and T3); as differences in FRS among accessions within each groups were observed more drastically in T3. Due to the low amount of tissue that could be collected mainly in the sensitive genotypes, RNA had to pooled equimolecularly to obtain enough high quality RNA for sequencing for some temperature/genotype combinations. A total of 34,075 genes were identified among all samples after RNA sequencing. The first approach was to obtain a general picture of the gene expression among temperatures and genotypes through a Principal Component Analysis (PCA). A total of 17,588 common genes expressed in the four genotypes, at the two developing stages and two temperatures were used to implement the PCA (Fig. 5). Two groups could be defined: one group included all the fruit samples and the other group the ovary samples, independently of temperature and genotype. A small variation between genes expressed was observed for the ovary samples.

The developing fruit samples were separated by both PC1 and PC2, accounting for 23.5% and 20.2% of the total gene variance, respectively. The samples collected in T2 were placed in the left area of the PC space (with negative values for PC1 and positive values for PC2), while most fruit samples sampled in T3 were located on the right area (with positive values for PC1 and negative values for PC2), reflecting the differential gene expression between temperature regimes (Fig. 5). On the other hand, no clear differences were observed between tolerant and sensitive genotypes with this analysis.

These results suggest that the response to heat stress induced more differential gene expressions between the analyzed genotypes in the early developing fruit stage than in the ovaries.

A) ***Differential expression analysis between modern cultivars LA2661 and LA2660***
  In order to obtain a deeper insight into the heat stress responses of tolerant and sensitive tomato varieties, the analysis was performed individually for each tomato cultivar group. According with the results from the PCA analysis, the higher gene expression differences among cultivars were observed among developing fruit samples. Thus, the subsequent analysis focused on those samples.
  In the case of the modern cultivars, the differentially expressed genes (DEG) within each variety, LA2661 and LA2660, between the T2 and T3 conditions, and between LA2661 and LA2660 in T3, were assessed at |log2 (fold-change)|≥ 2; p-value < 0.01.
  A total of 904 genes, 301 down-regulated and 603 up-regulated, were differentially expressed within the heat sensitive LA2660, between T2 and T3. The GO term enrichment analysis indicated a significant enrichment in biological processes related with the response to stress, including the response to heat and the response to temperature stimulus, which were enriched 15.42 and 13.21-fold respectively (Supplementary Fig. 5A).
  In the case of the DEG within the heat tolerant LA2661, between T2 and T3, 1411 DEGs were identified, including 645 down-regulated and 766 up-regulated genes. The GO term analysis indicated a significant enrichment in biological processes mainly related with regulation and photosynthesis. Also, the response to abiotic stimulus showed a 3.28 fold enrichment (Supplementary Fig. 5B).
  To better understand the common and specific responses of this group to heat, a DEG analysis was conducted between LA2660 and LA2661 in T3. A total of 1652 genes were differentially expressed between both genotypes; 927 of them were down-regulated and 725 up-regulated. The GO term categories response to heat and response to temperature stimulus were found to be significantly enriched 7.72 and 7.73 fold, implying 11 and 14 genes, respectively (Table 8, Supplementary Fig. 6). The response to heat stress of these cultivars included Heat Shock Proteins, overexpressed in the sensitive genotype LA2660, and three genes coding for hormones: *Solyc02g062390* (abscisic acid and environmental stress inducible protein) and enzymes: *Solyc06g059990* (alkyl-transferase) and *Solyc08g00568* (dimethylallylcistransferase, chloroplastic).

**Table 8:** Enriched genes for each biological process from the GO terms analysis, grey highlights the genes involved in the response to temperature. A minus symbol (-) means overexpressed for LA2660 (HS) and plus symbol ( +) overexpressed for LA2661 (HT)

	**protein complex oligomerization**	**response to hydrogen peroxide**	**response to abiotic stimulus**	**response to temperature**	**Family/subfamily**	**Protein class**	**Gene Expression**
Solyc01g102960	✓	✓	✓	✓	21.9 KDA HEAT SHOCK PROTEIN (PTHR11527:SF135)	chaperone	-
Solyc02g093600	✓	✓	✓	✓	17.6 KDA CLASS I HEAT SHOCK PROTEIN 3-RELATED (PTHR11527:SF316)	chaperone	-
Solyc03g113930	✓	✓	✓	✓	22.0 KDA CLASS IV HEAT SHOCK PROTEIN-LIKE (PTHR11527:SF271)	chaperone	-
Solyc06g076570	✓	✓	✓	✓	17.6 KDA CLASS I HEAT SHOCK PROTEIN 1-RELATED (PTHR11527:SF305)		-
Solyc09g015000	✓	✓	✓	✓	17.8 KDA CLASS I HEAT SHOCK PROTEIN-LIKE (PTHR11527:SF278)	chaperone	-
Solyc09g015020	✓	✓	✓	✓	17.8 KDA CLASS I HEAT SHOCK PROTEIN-LIKE (PTHR11527:SF278)	chaperone	-
Solyc12g042830	✓	✓	✓	✓	26.5 KDA HEAT SHOCK PROTEIN, MITOCHONDRIAL (PTHR11527:SF296)	chaperone	-
Solyc02g062390			✓	✓	ABSCISIC ACID AND ENVIRONMENTAL STRESS-INDUCIBLE PROTEIN TAS14-LIKE (PTHR33346:SF18)	-	+
Solyc03g007890			✓	✓	HEAT SHOCK PROTEIN 90–1 (PTHR11528:SF111)	Hsp90 family chaperone	-
Solyc06g059990			✓	✓	ALKYL TRANSFERASE (PTHR10291:SF16)	acyltransferase	+
Solyc07g040680			✓	✓	HEAT STRESS TRANSCRIPTION FACTOR A-9 (PTHR10015:SF298)	winged helix/forkhead transcription factor	-
Solyc08g005680			✓	✓	DIMETHYLALLYLCISTRANSFERASE, CHLOROPLASTIC (PTHR10291:SF22)	acyltransferase	-
Solyc08g062960			✓	✓	HEAT STRESS TRANSCRIPTION FACTOR A-2 (PTHR10015:SF338)	winged helix/forkhead transcription factor	-
Solyc12g007070			✓	✓	HEAT STRESS TRANSCRIPTION FACTOR C-1 (PTHR10015:SF332)	winged helix/forkhead transcription factor	-
Solyc01g079200			✓		GIBBERELLIN 2-BETA-DIOXYGENASE 6 (PTHR47990:SF45)	-	-
Solyc01g091430			✓		X-RAY REPAIR CROSS-COMPLEMENTING PROTEIN 5 (PTHR12604:SF4)	-	+
Solyc03g006880			✓		GIBBERELLIN 20 OXIDASE 1 (PTHR47990:SF53)	-	-
Solyc03g112920			✓		OS03G0310200 PROTEIN (PTHR34946:SF2)	-	+
Solyc05g055020			✓		PROTEIN LIGHT-DEPENDENT SHORT HYPOCOTYLS 1 (PTHR31165:SF70)	-	-
Solyc06g035530			✓		SUBFAMILY NOT NAMED (PTHR47990:SF113)	-	+
Solyc06g066820			✓		GIBBERELLIN 3-BETA-DIOXYGENASE 1 (PTHR47990:SF73)	-	+
Solyc06g072360			✓		PROTEIN INDETERMINATE-DOMAIN 16-LIKE (PTHR34946:SF11)	-	+
Solyc09g074530			✓		BIDIRECTIONAL SUGAR TRANSPORTER SWEET (PTHR10791:SF157)	-	+
Solyc11g068620			✓		NAC DOMAIN-CONTAINING PROTEIN 90 (PTHR31989:SF4)	-	+
Solyc12g006140			✓		CHLOROPHYLL A-B BINDING PROTEIN, CHLOROPLASTIC (PTHR21649:SF100)	-	-
B) ***Differential expression analysis between “de penjar/da serbo” genotypes, TRVI0040 and TRBA0160***
  An analysis of differentially expressed genes was conducted for TRVI0040 and TRBA0160 between T2 and T3, and between both of them in T3 in developing fruit samples.
  A total of 773 genes were differentially expressed in TRBA0160 between T2 and T3, 592 were down-regulated and 181 were up-regulated. The GO term analysis indicated a significant enrichment of biological processes mainly associated with metabolism (Supplementary Fig. 7A). In the case of the heat tolerant TRVI0040, 3364 differential expressed genes were down regulated and 931 upregulated. The biological processes enriched in this genotype were more diverse, involving genes associated with photosynthesis, regulation and with response to different stresses: response to stress (3.08 fold), response to abiotic stimulus (2.32 fold), response to stress (1.76 fold) and response to stimulus (1.81 fold) (Supplementary Fig. 7B).
  Moreover, the comparison between TRVI0040 and TRBA0160 in T3 revealed 1821 differentially expressed genes, of which 709 were down-regulated and 1112 were up-regulated. The GO term analysis of the biological processes mainly showed the enrichment of genes involved in metabolic processes and did not include enrichment of genes that were directly associated to high temperature stress (Supplementary Fig. 8).
  The analysis of the genes that were overexpressed in the different categories for biological processes showed 81 genes that were involved in the response to stimulus. In this category, 28 genes belonged to families related with response to stress direct or indirectly. Of these, six genes were involved in heat response and three of them were common with the modern tomato cultivars group (Table 9).

**Table 9:**
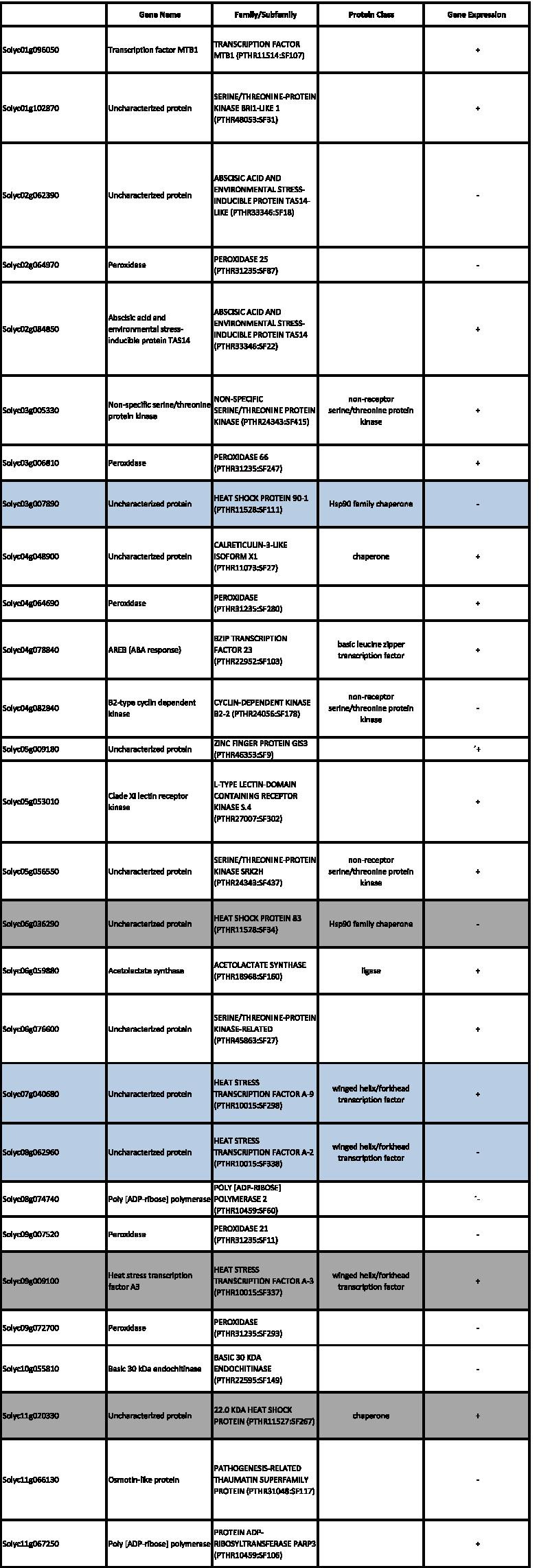
Enriched genes associated with response to stress for biological processes from the GO terms analysis, grey highlights the specific genes involved in the response to temperature and blue indicates the common enriched genes as compared with the modern cultivar genotypes. A minus symbol (-) means overexpressed in TRBA160 (heat sensitive) and a plus symbol ( +) overexpressed for TRVI0040 (heat tolerant)
  Thus, the response to heat of the “de penjar”/”da serbo” tomatoes activated 29 genes involved in different biological processes, only five of them belonging to the HSP family, and *Solyc06g036290* (overexpressed in the heat sensitive TRBA160), *Solyc09g009100*, and *Solyc11g020330* (both overexpressed in TRVI0040 heat tolerant) were enriched specifically in these tomato group genotypes as compared with the modern cultivars.
C) ***Comparison of gene expression between different groups***

The analysis described above suggested different high temperature response mechanisms for each tomato group. The developing fruit DEG analysis revealed variability in the genes activated between genotypes, independently of their assignment to a cultivar group. The comparison of DEG between T2 and T3 for the two heat sensitive genotypes, LA2660 and TRBA160, showed enrichment of genes involved mainly in metabolic processes in both temperature regimes (Supplementary Figs. 5A and 7A). On the other hand, the DEG for LA2660 were involved in regulation of enzymes and response to heat and temperature stimulus (Supplementary Fig. 5A). In the case of the tolerant genotypes, differences in DEG were also evident, with LA2661 showing enrichment in genes involved in physiological processes (Supplementary Fig. 5B), and TRVI0040 in response to stress (Supplementary Fig. 7B). The comparison between the contrasting genotypes within each group in T3 confirmed the different responses, with the enrichment of genes in response to stress, including heat and temperature in the LA2660 *vs* LA2661 comparison (Supplementary Fig. 6), and the enrichment of genes associated to response to hormone and endogen stimulus for TRVI0040 and TRBA160 (Supplementary Fig. 8). Moreover, three genes were differentially expressed in both comparison (LA2660 *vs* LA2661 at T3, and TRBA160 and TRVI0040 at T3). *Solyc03g007890* and *Solyc06g062960* were overexpressed in the heat sensitive LA2660 and TRBA160, and the expression of *Solyc07g040680* showed differences in the expression pattern, being overexpressed in LA2660 (HS) and in TRVI0040 (HT).

Lastly, in order to find the common genes that played a role in the response to heat in the two tomato groups, a comparison between the responses in T3 of the tolerant genotypes was performed. A total of 1354 DEG were observed between LA2661 and TRVI0040 in T3. Of these, 825 were expressed higher in LA2661, while the remaining and 529 were expressed higher in TRVI0040. Sixteen of those differentially expressed genes were involved in heat tolerance (Table 10).

**Table 10:**
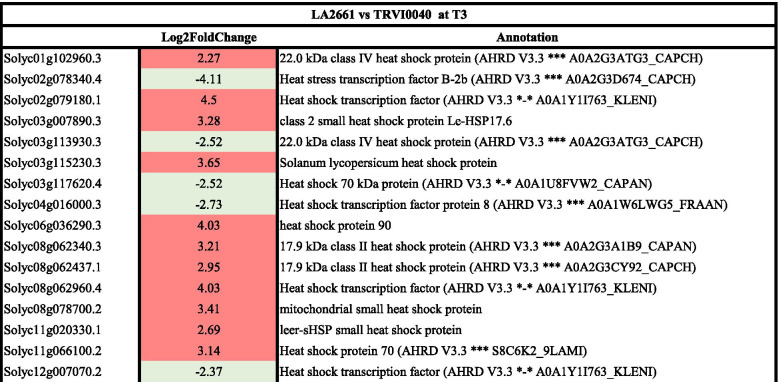
Differentially expressed genes between the tolerant accessions LA2661 and TRVI0040 at T3. A negative number indicates overexpression in LA2661, and positive numbers overexpression in TRVI0040

Moreover, the GO term analysis revealed the enrichment in only the biological processes related to response to heat stress: response to abiotic stimulus (3.95 fold), response to heat (7.18 fold), and response to temperature stimulus (8.38 fold) (Supplementary Fig. 9A). From the 24 genes enriched for biological process, nine were involved in response to heat (*Solyc01g102960.3*, *Solyc03g007890.3*, *Solyc03g113930.3*, *Solyc03g115230.3*, *Solyc06g036290.3*, *Solyc08g062340.3*, *Solyc08g062960.4*, *Solyc11g020330.1*, *Solyc12g007070.2*), corresponding with differentially expressed genes between both genotypes in T3 (Table 10). The GO enrichment for molecular functions showed two functions: hydrolase activity, hydrolyzing O-glycosyl compounds enriched 3.96 fold, and chitinase activity with an enrichment of 1.47 fold (Supplementary Fig. 9B).

Comparing the DEG between pairs of cultivars within each cultivar group, some DEGs were uniquely observed within one of the cultivar groups (Table 11). In the case of the modern varieties, the DEG between LA2661 and LA2660 in T3 included three differentially expressed genes specifically between these cultivars (*Solyc02g062390*, *Solyc06g059990*, and *Solyc08g005680*). Regarding the DEGs between the “de penjar/ da serbo” cultivars, *Solyc09g009100* was exclusively differentially expressed in this group. The last comparison including the heat tolerant genotypes from the two groups showed four genes that were exclusively differentially expressed between LA2661 and TRVI0040 in T3 (*Solyc01g102960.3*, *Solyc03g113930.3*, *Solyc03g115230.3*, and *Solyc08g062340.3*). On the other hand, two common genes for the three comparisons and four that were common in at least two of the comparisons were identified (Table 11).

**Table 11:** List of genes related with heat stress that were differentially expressed in T3 in each comparison of the contrasting genotypes from each tomato group (LA2661 vs LA2660 T3, and TRVI0040 vs TRBA160 T3), and the comparison against the heat tolerant genotypes from the two groups (LA2661 vs TRVI0040 T3). The genotype in which the gene was highly expressed appears on the table

	**Family/Subfamily**	**Comparisons**		
		**LA2661 vs LA2660 T3**	**TRVI0040 vs TRBA160 T3**	**LA2661 vs TRVI0040 T3**
Solyc03g007890	HEAT SHOCK PROTEIN 90–1 (PTHR11528:SF111)	LA2660	TRBA160	TRVI0040
Solyc08g062960	HEAT STRESS TRANSCRIPTION FACTOR A-2 (PTHR10015:SF338)	LA2660	TRBA160	TRVI0040
Solyc01g102960.3	22.0 kDa class IV heat shock protein (AHRD V3.3 *** A0A2G3ATG3_CAPCH)	LA2660		TRVI0040
Solyc03g113930.3	22.0 kDa class IV heat shock protein (AHRD V3.3 *** A0A2G3ATG3_CAPCH)	LA2660		LA2661
Solyc06g059990	ALKYL TRANSFERASE (PTHR10291:SF16)	LA2660		TRVI0040
Solyc08g005680	DIMETHYLALLYLCISTRANSFERASE, CHLOROPLASTIC (PTHR10291:SF22)	LA2660		LA2661
Solyc12g007070	HEAT STRESS TRANSCRIPTION FACTOR C-1 (PTHR10015:SF332)	LA2660		LA2661
Solyc07g040680	HEAT STRESS TRANSCRIPTION FACTOR A-9 (PTHR10015:SF298)	LA2660	TRVI0040	
Solyc11g020330	22.0 KDA HEAT SHOCK PROTEIN (PTHR11527:SF267)		TRVI0040	TRVI0040
Solyc06g036290	HEAT SHOCK PROTEIN 83 (PTHR11528:SF34)		TRBA160	TRVI0040
Solyc02g062390	ABSCISIC ACID AND ENVIRONMENTAL STRESS-INDUCIBLE PROTEIN TAS14-LIKE (PTHR33346:SF18)		TRBA160	
Solyc09g009100	HEAT STRESS TRANSCRIPTION FACTOR A-3 (PTHR10015:SF337)		TRVI0040	
Solyc03g115230.3	Solanum lycopersicum heat shock protein			TRVI0040
Solyc08g062340.3	17.9 kDa class II heat shock protein (AHRD V3.3 *** A0A2G3A1B9_CAPAN)			TRVI0040

Eleven DEGs belonged to the HSPs family. Of these, *Solyc03g007890* and *Solyc08062960* were differentially expressed between T2 and T3 temperature regimes for both groups: overexpressed in the heat sensitive genotypes, and in LA2661 in the comparison between LA2661 vs TRVI0040. *Solyc06g059990*, coding for an alkyl transferase, and *Solyc08g005680* for a dimethylallylcistransferase chloroplastic, were differentially expressed between LA2661 and LA2660, LA2661 and TRVI0040, both in T3. Also, Solyc02g062390 was overexpressed in TRBA160 as compared with TRVI0040 in T3.

These results suggest the occurrence of common responses to high temperature stress combined with differential responses to heat in each group. All the genotypes induced genes related with heat stress, heat shock proteins, or heat transcription factors. Moreover, along with the activation of HSPs, a differential expression of genes that regulate hormones or enzymes, such abscisic acid and transferases, was found.

## Discussion

The heat tolerance of a large number of tomato accessions from a wide range of different genetic materials was tested to find sources of genetic tolerance to high temperatures. The selected materials will be a precious source in breeding programs aimed at developing heat-tolerant varieties and also to understand the genetic control of the tolerance mechanisms. The combination of phenotyping in controlled greenhouse conditions and open field trials allowed observing the response of selected genotypes under a wide range of heat stress conditions.

The initial screening of 219 accessions revealed that in general, FLN was not importantly reduced in moderate heat stress (T2), although it was heavily affected in high stress (T3). Previous reports also showed that in moderate heat stress (32 °C day/28 °C night), FLN decreased slightly [47, 48], supporting our observations, whereas at extreme high temperatures (38 °C day/ 28 °C night), a strong decrease in FLN was also observed [49]. Therefore, in general, tomatoes seem to be capable of developing flowers under moderate heat stress but not under extreme heat stress, except for a relatively few number of genotypes.

In the case of FRN, in the current study, both moderate and extreme heat stress caused its decrease, with important differences between the genotypes tested. Similarly, previous studies showed the impact of heat stress on FRN [22, 25, 44, 48].

The reduction in FRS under heat stress is one of the common indexes used to determine the heat tolerance both in greenhouse [46, 48, 50, 51] and open field experiments [29, 44]. The negative effect of high temperatures on FRS in tomato and the correlation of this trait with yield [18], confirm FRS as one of the main discriminating factors that could be utilized to assess the tolerance to high temperatures. Based on the FLN and FRN results, the calculation of FRS provides us with a better estimation of the tolerance of the analyzed accessions. Thus, in the first screening of the accessions (FCCV_2016), a high FRS variability was observed under heat stress. Moreover, 35% of the genotypes had significantly higher FRS under high temperatures than the sensitive control ‘MoneyMaker’. The genotypes with better performances belonged to the group of modern varieties, either modern cultivars or commercial varieties with 42% and 68% of the accessions with good FRS, respectively. The next group with a higher number of accessions which showed a good response to high temperatures was the traditional “de penjar/da serbo” accessions, with 33% of them showing an improvement as compared to the control ‘MoneyMaker’. We observed different responses to heat stress: a gradual decrease together with the temperature increase, and tolerance only in the extreme temperature regime, suggesting an adaptation to the environmental conditions. Acclimation is a reversible form of plasticity in response of environmental variation [52].

The augmented design allowed us to screen a large germplasm collection, thereby providing a potentially good balance between massive screening and discriminative power. To assess it, the candidate heat tolerant genotypes were evaluated in replicated trials to confirm their tolerant response. Thus, the selected wild species accessions showed statistically significant heat tolerance in replicated trials (FCCV_2017 and ENZA_2018 experiments). In FCCV_2017, the eight accessions tested showed high FRS values in T2, whereas 75% (5 accessions) were superior to the sensitive control ‘MoneyMaker’ for FRS (Table 3). In the case of the ENZA_2018 experiment, the FRS for the three wild accessions analyzed was lower in T2 and T3 as compared with the previous results in the FCCV_2016 and FCCV_2017 experiments (Table 4). The ‘Monterrey’ control also showed a decrease in FRS, compared with the other greenhouse experiments, perhaps due to the different style of agronomic management with longer heat treatments and harvesting at an optimal ripening stage, which could have also affected to the FRS of selected wild accessions. Nevertheless, their response to heat stress was similar to ‘Monterrey’. The negative effect of heat stress on fruit ripening, with production of parthenocarpic or low-quality fruits [47, 53] discarded in commercial assays, could have contributed to the final FRN and, consequently, to the decrease in FRS. The use of wild tomatoes as sources of tolerance to heat stress have already been reported [18], although the polygenic nature of these complex traits makes difficult the recovery of the agronomic advantage of elite lines after their introduction into breeding programs [54].

Regarding the 23 *S. lycopersicum* accessions selected from the FCCV_2017 experiment, 16 of them showed statistically significant heat tolerances in either T2 or T3, or both in the replicated trial (FCCV_2017). Therefore, 70% of the *S. lycopersicum* accessions selected, based on an augmented design experiment, demonstrated heat tolerance in replicated trials. Taking both *S. lycopersicum* and wild accessions together, the heat tolerance of 77% of them was verified. The accessions assessed in the ENZA_2018 experiment also showed similar levels of tolerance as the control ‘Monterrey’. A lack of verification may due to false positives from the augmented design, although false negatives could have also been found. Nevertheless, the ratio of success can be considered high, supporting the use of an augmented design for large-scale screening of heat tolerant genotypes.

Regarding the varieties provided by the UNINA and the MVCRI, based on heat tolerance in the field, most UNINA accessions showed a certain level of heat tolerance in the FCCV_2017 experiment. On the other hand, heat tolerance was observed only in one of the MVCRI varieties (‘BG ALIA’). These varieties are adapted to different pedoclimatic areas (Mediterranean, Central Europe). The greenhouse heat stress conditions may have closely resembled the Mediterranean weather, for example for the duration in time of the high temperatures, what would explain their better behavior in the FCCV_2017 trial. In fact, the complexity of the response to heat stress and the influence of multiple factors in the tolerance to this abiotic stress is well documented [55].

The verification of heat tolerance in field experiments was a further step for evaluating their value for breeding programs. The importance of this verification in natural heat stress conditions was already reported in tomato [29] with a small number of samples, with most of the accessions selected in the greenhouse also showing a good response to high temperatures in open fields. Moreover, the shift from experiments in the greenhouse under controlled conditions to field experiments included the effect of other environmental variables including light or water depletions [56] which could have been the cause of the differences in the response to heat stress of some of the genotypes.

Eight varieties showed a high FRS under high temperatures in both greenhouse and field experiments: one *S. lycopersicum* var. *cerasiforme* (BGV006071), three modern F_1_ hybrids (‘Durinta’, ‘PaiPai’, ‘TEMPTATION’), and four Italian landraces (E7, E8, E36 and E37). E76 did not show heat tolerance either in the greenhouse or the field experiment. The only discrepant behavior was found for BG1923/15, with good heat tolerance in the field but no tolerance in the greenhouse experiments. As stated before, this discrepancy could be attributed to a different adaptation to heat stress conditions. This high success ratio in the similar stress response between greenhouse and field experiments was already obtained, although with a radically different screening strategy [29] which relied on the selection on based chlorophyll fluorescence traits, whereas in the current report we relied on FRS and a larger germplasm screening. Nevertheless, both works encourage the strategy of preliminary screening in controlled conditions for selecting genotypes with a high potential for heat tolerance in the field.

The physiological basis of the heat tolerance of these eight varieties still needs to be investigated. The photosynthesis efficiency could be involved in the heat tolerance of tomato cultivars [29, 57]. The photosynthesis efficiency under heat stress in several tomato cultivars, including E7, E8 and E37 has already been studied [28], although a clear pattern of photosynthesis efficiency parameters were not observed between these cultivars that could be related to their heat tolerance. Pollen viability has been found to be strongly correlated with fruit set at high temperatures [47, 57, 58]. In the current experiments, pollination was not monitored, so in the case that one variety would have had a higher viability, pollen from this variety could have pollinated other varieties, and no contrasting phenotypes could have been observed. Other traits that have been proposed as components on heat tolerance in terms of fruit set are female fertility, accumulation of different metabolites (sugars, proline transporters, polyamines, flavonoids, etc.), control of canopy temperature, and membrane stability [22]. Given the wide genetic basis investigated in the current report, different mechanisms may be involved in the observed heat tolerance.

The varieties and accessions studied in the current work have shown differences depending on the experimental conditions (two greenhouses, two fields) but, in general, the selected genotypes were able to set fruits in all the growing conditions assayed. The plasticity in the crop’s response to multi-environment assays provides a better understanding of the mechanisms utilized to overcome a given stress [59]. The heat tolerance of the accessions in different environments could be due to the plasticity in metabolic reactions as a response to unfavorable environments [60], and could also be part of a general response to multiple stresses [61], as reported in other species such as cereals [32, 62, 63]. In tomato, recent studies have highlighted the complex genetic basis of phenotypic plasticity and genotype x environment interactions when facing a climate change scenario [34].

In summary, eight varieties showed heat tolerance in both field and greenhouse experiments and one variety (BG1923/15) showed tolerance in the field. Additionally, eight wild species accessions (BGV007109, LA0480, BGV007947, BGV008114, BGV007111, BGV008030, LA2147, and LA2184), the *S. lycopersicum* var. *cerasiforme* BGV012641, four traditional varieties (TRVA0030, TRVIO040, BGV007932, and BGV004582), one F_1_ hybrid (‘Vento’), and one modern inbred (‘BG ALIA’), were found to be heat tolerant in replicated greenhouse experiments. Therefore, a total of 24 tomato genotypes from diverse origins and different tolerant responses to heat stress are reported. Thus, this report fills, at least in part, the gap from previous reports where screening for heat tolerance was carried out only in a limited number of cultivars [16, 44, 46, 64–66].

Furthermore, the gene expression analysis of contrasting genotypes for the tolerance to heat from two different groups (modern cultivars and “de penjar/ da serbo” accessions), showed differences in the response to high temperature at the transcriptional level. The differences observed were mostly found in fruits in the button stage (post-anthesis), whereas the ovary samples (pre-anthesis) showed a similar response between themselves independently of temperature, tomato group, and sensitive or tolerant genotype. Differences depending on the tissue and the developmental stage have already been reported by other authors [8, 53, 67–69]. However, the reproductive phases have been described to be more sensitive to high temperatures, and affecting both male and female organs [70], although the ovules were found to be generally less heat sensitive than pollen [71]. Unfortunately, the lack of samples from the anthers in our experiments, due to the damage in this tissue by the high temperature treatments in our experimental conditions, negatively affected the availability of transcriptomic information from our experiments. On the other hand, the genes identified in the DEG analysis, in fruit samples depending on temperature and genotype sensibility, as opposed to ovaries, indicated than in our conditions the response to heat took place in a late stage of development.

To gain a deeper insight into the response to heat stress, fruit DEGs were dissected by comparing each genotype according to group and temperature. The transcriptomic analysis of the fruit revealed differences in the response of the genotypes to heat stress. The complexity of the plants’ response to elevated temperatures and their variability have already been reported [38]. For our genotypes, the DEG analysis revealed an increase in the gene expression of HSP genes during early fruit development, especially for the genotypes from the modern cultivars group. The control of heat stress due to the accumulation of heat shock proteins is a common mechanism against this stress [39] and has been reported for different plant species as *Arabidopsis thaliana* [72–74], rice [75], wheat [76] or tomato [77, 78]. Among all the DEG enriched in the different comparisons carried out in this assay, a common response of two genes *Solyc03g007890* (coding for HSP 90) and *Solyc08g062960* (coding for the heat stress transcription factor A-2) was observed in the sensitive genotypes of both tomato groups. Both genes were overexpressed under heat stress in those genotypes. However, another gene also encoding for a heat stress transcription factor (A-9), *Solyc07g040680,* was enriched but with a different expression pattern in each group, as it was overexpressed in the heat sensitive LA2660 modern cultivar and in the heat tolerant TRVI0040 from the “de penjar/ da serbo” group. The low number of common genes in the response to heat in the different tomato groups suggests the need for a deeper analysis of each of them to decipher the mechanisms of heat tolerance.

Despite the main response of the HSPs, other mechanisms were induced as a response to heat stress in plants. Hormones such as ABA, transcription factors, or enzymes, essentially contribute to the mechanisms of response to high temperatures [74, 79]. In the current experiments, we also observed clear DEG related to these processes. The sensitive genotypes from modern cultivars showed an increase in the expression of HSP genes in their response, whereas in the tolerant ones, a high induction was observed in the expression of the ABA hormone and Alkyl transferase genes. The role of the key stress response hormone, ABA, in the tolerance to several abiotic stresses, together with other transcription factors, have been described as being essential for inducing the mechanisms underlying plant stress responses [80]. On the other hand, the high temperature response observed in the “de penjar /da serbo” group required the differential expression of genes coding for enzymes, hormones and transcription factors in a greater proportion than HSP genes. Among the genes that were overexpressed as a response to heat stress in these genotypes, we identified genes encoding enzymes such as serine/threonine kinase [81]; transcription factors from the BZip family involved in the response to heat and other stresses in *Arabidopsis thaliana* [82] and tomato [83]; or genes coding for peroxidases, usually activated under heat stress to fight against ROS (Reactive Oxygen Species) [84].

The complexity of the response to heat stress in tomato becomes evident from the results presented herein, depending on the genotype and the organ’s developmental stage. Differential responses between tolerant and sensitive genotypes were detected mainly in early developing fruit stages, with low or no differences at the ovary stage. A common response to heat stress between sensitive genotypes was the overexpression of genes encoding HSPs, whereas the response between tolerant genotypes involved genes from other biological categories. Elucidating the specific mechanisms of each tomato group to heat stress tolerance could provide useful tools for the development of new thermotolerant varieties for facing climate change.

## Conclusions

The large number of accessions from different tomato types screened in the present study, and the verification of their response to heat stress in different environments, allowed us to identify strong heat tolerance sources, and to provide a wide range of genetic materials to be included in breeding programs. A total of 24 tomato genotypes from diverse origins and different tolerant responses to heat stress under greenhouse controlled conditions were identified. Moreover, the heat tolerance of eight genotypes was verified in field conditions. Therefore, fruit set in high temperatures in controlled greenhouse experiments can be used as a predictor of field performance in high temperature stress.

Furthermore, the gene expression analysis revealed the complexity of the response to heat stress in tomato, which was dependent on the genotype and the organ’s developmental stage, with a major response to heat at the post-anthesis level. The main differential responses between sensitive and tolerant genotypes were observed in early developing fruit stages, with the overexpression of genes encoding HSPs in the response of sensitive genotypes and genes involved in different biological categories for tolerant genotypes.

## Methods

### Plant material and experiments

Two hundred-and-thirty five tomato genotypes were studied in the different experiments. These genotypes had very diverse origins: wild species accessions (*S. pimpinellifolium* and *S. cheesmaniae*), early domesticates (*S. lycopersicum* ssp*. cerasiforme*), traditional varieties, landraces, modern cultivars and commercial hybrids (Supplementary Table 1).

The plant material was provided by COMAV (Institute for the Conservation and Improvement of Valencian Agrodiversity. Valencia, Spain), EELM (Experimental Station “La Mayora”- Spanish National Research Council. Malaga, Spain), TGRC (Tomato Genetics Resource Center at UC Davis. California, USA), TRADITOM (Plant material from the European Union’s Horizon 2020 project TRADITOM), ENZA (ENZA ZADEN. Seed company. Almeria, Spain), MVCRI (Maritsa Vegetable Crops Research Institute. Plovdiv, Bulgaria), UNINA (University of Naples Federico II. Portici, Italy), and IBMCP (Institute for Plant Molecular and Cellular Biology_ Spanish National Research Council. Valencia Spain). The contribution of each institution to the experiments is detailed in Supplementary Table 1. The authors declare that all the experiments performed in this study complied with the institutional, national and international guidelines and legislation.

Six experiments were carried out including different combinations of the above genotypes (see Fig. 1 for a scheme of all experiments and Supplementary Table 1 for details on what accessions/varieties were included in each experiment):

#### FCCV_2016

Two hundred-and-nineteen genotypes were analyzed: 80 wild species accessions (79 *Solanum pimpinellifolium*, 1 *S. cheesmaniae*), 16 *S. lycopersicum* var. *cerasiforme* accessions, 48 traditional cultivars (36 of them belonging to the traditional groups “de penjar” and “da serbo”, from Spain and Italy, respectively, that are traditionally cultivated in dry and hot conditions) and 75 modern cultivars and commercial hybrids. Five controls were also included: the variety ‘MoneyMaker’, a Spanish traditional cultivar (TRVA2360), and the modern hybrids ‘Docet’, ‘Monterrey’ and ‘JAG8810’. The plants were cultivated on drip fertigation inert substrate bags in a greenhouse under controlled temperatures in the facilities of the Centro de Experiencias Cajamar (FCCV, Paiporta, Spain) in the spring–summer season. The genotypes were distributed into six blocks corresponding with the greenhouse rows following an augmented design with a single replicate per tested genotype, six replicates for ‘MoneyMaker’, ‘Monterrey’, and TRVA2360, four for ‘Docet’, two for ‘JAG8810’ (the lower number of replicates was due to limitations in the plant material) per block. Replicates consisted of three plants cultivated in a single bag. The plants were grown under a stepwise temperature increase, ensuring a minimum temperature in each regime (T1: 25 °C day/20 °C night; T2: 30 °C day/25 °C night; T3: 35 °C day/30 °C night) as previously described [14]. Each temperature regime was set for 4 weeks.

#### FCCV_2017

Forty one candidate heat tolerant accessions and varieties were studied in this experiment. Plants were cultivated in the FCCV facilities following the same agronomic management and temperature regimes as the previous FCCV_2016 experiment. The greenhouse was divided into two blocks, separating the wild species accessions and the cultivars due to the different plant phenology between these two groups. Five replicates (three plants grown in a single bag) per accession/cultivar were randomly distributed into each block, with eight replicates for the controls ‘MoneyMaker’ and ‘Monterrey’.

#### ENZA_2018

This experiment was carried out in the facilities at the Enza Zaden Centro de Investigación S.L. (Almería, Spain). Fourteen selected heat tolerant candidate varieties were grown in a greenhouse under a stepwise temperature increase (T1: 30 °C day/18 °C night; T2: 35 °C day/25 °C night; T3: 40 °C day/28 °C night) in semi-controlled temperature conditions (i. e. the minimum temperatures could be controlled and maintained throughout the temperature regimens, but the maximum temperatures could occasionally be higher depending on external climatic conditions), for five weeks for each temperature regime. Plants were grown according to standard commercial agronomic management, i. e., fruit were not pruned during the experiment and harvested at full ripening. The same plants were used for each temperature regime, as in the FCCV experiments, with four replicates per accession and the control ‘Monterrey’.

#### MVCRI_2018

The experiment was carried out in the experimental fields at Maritsa Vegetable Crops Research Institute (Plovdiv, Bulgaria). Sixteen heat tolerant candidate varieties were grown following a completely randomized design with two replicates per variety. ‘MoneyMaker’ was used as a heat sensitive control with two replicates as well.

#### UNINA_2018

Eleven heat tolerant candidate varieties were cultivated following a randomized design with three replicates per accession and three plants per replicate. The plants were grown in an experimental field in the region of Campania (Italy), at the facilities of the University of Naples Federico II, ‘MoneyMaker’ and ‘Monterrey’ were used as the heat sensitive and tolerant controls, respectively, with three replicates per accession and three plants per replicate.

#### RNASeq_2019

Two pairs of varieties belonging to the same horticultural group each TRBA0160 and TRVI0040 (‘de penjar’ or ‘da serbo’ traditional tomatoes), LA2660 and LA2661 (modern varieties) with contrasting responses to heat stress (sensitive TRBA0160 and LA2660, tolerant TRVI0040 and LA2661), were selected for the differential gene expression analysis in 2019. The culture management was similar to the FCCV_2016 and FCCV_2017 experiments, with a stepwise temperature increase (T1: 25 °C day/20 °C night; T2: 30 °C day/25 °C night; T3: 35 °C day/30 °C night). Replicates consisted of three plants cultivated in a single bag with three replicates per accession.

### Phenotyping

#### FCCV_2016 and FCCV_2017

During the third week of each temperature regime, the number of flowers (FLN) in the second and third truss was recorded. The number of fruit (FRN) was recorded on the fourth week from the same trusses. The fruit set (FRS) ratio was calculated as follows: FRS = 100*FRN/FLN. Before each temperature regime change, flowers and fruit were pruned from each plant in order to avoid the physiological effects of previous fruit load on the new inflorescences. Pollination was ensured by incorporating bumblebees as an external pollinator to greenhouses.

#### ENZA_2018

On the fifth week of each temperature treatment, the number of flowers (FLN) was recorded from all the trusses produced at the corresponding temperatures. The trusses were labeled to indicate at what temperature regime they were produced in and to record the FRN corresponding to each temperature regime. The fruits were harvested at the optimal ripening stage according to standard commercial harvesting guidelines. FRS was calculated as before. Pollination was facilitated by vibrating the plants three times a week. **MVCRI_2018:** The flower number was recorded starting from the second truss to the fifth truss (trusses 2, 3, 4, and 5). The day/night temperatures were recorded each week, and a mean temperature was assigned to each truss. The number of fruits was recorded after harvesting at the optimal ripening stage. Harvested fruits were used to calculate the FRN and FRS.

#### UNINA_2018

Tomato plants were grown following the standard cultural practices of the area. FLN and FRN were evaluated on inflorescences produced on the second to the fifth truss. FRS was calculated after the harvest at the optimal commercial stage. The temperature was measured during the entire growing season.

#### RNASeq_2019

At each temperature treatment, the flower and fruit number were counted as described in the previous FCVV experiments. FLN an FRN were recorded on the third and the fourth week of each treatment, respectively. Moreover, pre-anthesis ovaries and developing fruit (about 1 cm in size) were sampled for RNA extraction.

### Statistical analysis

An augmented design was implemented in the FCCV_2016 experiment. The adjusted factor (R) for each block was calculated with the equation

$$Rj=(\mathrm{B}\mathrm{j}-\mathrm{M})/\mathrm{c}$$

where B_j_ was the sum of values for all the controls in block j; c was the number of controls and M the sum of the means of all the controls. The FLN, FRN and FRS values for the tested genotypes were recalculated using the adjusted factor for each block.

The minimum significant difference (MSD) was calculated from the controls’ variance difference (S) using the equations:

$${\mathrm{S}}_{vc}^{2}=\mathrm{C}\mathrm{M}\mathrm{E}(\mathrm{b}+1)(\mathrm{c}+1)/\mathrm{b}\mathrm{c}$$

$$\mathrm{M}\mathrm{S}\mathrm{D}=\mathrm{t}(0.05)\surd {\mathrm{S}}_{\mathrm{v}\mathrm{c}}^{2}$$

where CME was the Control mean square error, b the number of blocks and c the number of controls. In the MSD equation, t corresponds to the t value for [(b-1)/(c-1)] degree of freedom.

The values of the controls and genotype were adjusted based on these equations, and the threshold for significance was calculated based on the adjusted values.

For all the experiments, the basic statistics (Mean, standard deviation, maximum value and minimum values), and Pearson’s correlations were calculated.

In all the experiments, except for FCCV_2016, the FRS means of tested genotypes were compared with the respective controls with a Dunnett’s test at *p *< 0.05. Moreover, the genotypes were ranked with the multiple mean comparison Tukey’s test at *p *< 0.05.

The genotype, temperature effects and their interaction were studied for the control varieties of the FCCV_2016 experiment by two-way ANOVA. Likewise, the common genotypes evaluated in the FCCV_2017 and ENZA_2018, genotype x environment interactions were also analyzed by with a two-way ANOVA:

$${\mathrm{Y}}_{\mathrm{i}\mathrm{j}\mathrm{k}}=\mathrm{\mu }+{\mathrm{G}}_{\mathrm{i}}+{\mathrm{E}}_{\mathrm{j}}+{\mathrm{G}\mathrm{x}\mathrm{E}}_{\mathrm{i}\mathrm{j}}+{\mathrm{e}}_{\mathrm{i}\mathrm{j}\mathrm{k}},$$

where Y_ijk_ is the value of the kth replicated of the ith genotype (G) in the jth environment (E), GxE is the interaction, and e_ijk_ the error.

For FCCV_2016, G_i_ corresponds to the four control varieties and E_j_ to the three temperature regimes (T1, T2 and T3). Regarding FCCV_2017 and ENZA_2018, G_i_ corresponds to the common genotypes and E_j_ to the different experiments.

All the statistical analysis were performed with the JMP 12.1.0 software [85].

### RNASeq_2019

Tissue from two organs at different developmental stages, pre-anthesis (ovary) and post-anthesis (fruit at the button stage) from plants grown in T2 and T3 were selected for RNASeq analysis. The RNA isolation was performed using the NucleoSpin RNA II kit (Macherey–Nagel, Germany) for each sample. The amount of RNA was measured with the NanoDrop™ 2000/2000c spectrophotometer (Thermo Scientific™, USA). Three independent samples, one for each replicate, with a total RNA concentration of at least 50 ng/uL were sent to Macrogen, Inc (Republic of Korea) for RNA-Seq analysis.

### Transcriptome analysis

From the total RNA, mRNA libraries were generated using TruSeq Stranded technology (Ilumina, USA). RNA sequencing was performed with the NovaSeq platform, 150 bp paired-end reads, 6 Gb/sample (Ilumina, USA). High quality RNA, with RIN (RNA integrity number) >  = 7.5, was obtained for the fruit samples in T2 for LA2660, LA2661, TRVI0040 and TRBA160, ovaries in T2 for LA2660, TRBA160, and ovaries in T3 LA2661, LA2660, TRVI0040, and TRBA160. These samples were sequenced for each replicate individually. For the other experimental samples, the quantity of RNA obtained was not sufficient for sequencing, so they were pooled. Thus, the comparison for developing fruit samples in T2 LA2661 vs LA2660, LA2661 in T2 vs LA2661 in T3, LA2660 in T2 vs LA2660 in T3, TRVI0040 vs TRBA160 in T2 and TRBA160 in T2 vs TRBA160 in T3 were performed with the replicated samples. The rest of the comparisons were performed with the sequences of the pooled samples. The sequences were aligned against the SL 4.0 tomato genome. The read counting, adscription of read number to each gene was performed with htseq-count (https://htseq.readthedocs.io/en/release_0.11.1/count.html). The differential expression analysis of genes was performed with DESeq2 (http://bioconductor.org/packages/release/bioc/vignettes/DESeq2/inst/doc/DESeq2.html). The results were presented as a RPKM (Reads Per Kilobase Million) and the LFC ratio (log2 fold change). The threshold used to identify differentially expressed genes was set at LFC ≥|2| and *p*-value < 0.01. A gene was defined as expressed when it obtained a RPKM greater than zero in at least one sample. The principal component analysis was performed using ClustVis web tools (http://biit.cs.ut.ee/clustvis/).

Gene ontology (GO) categories and enrichment analysis of the differentially expressed genes were found using the online ‘The gene ontology resource’ (http://geneontology.org/). Overexpressed GO categories were found using the PANTHER Classification System (http://pantherdb.org/webservices/go/overrep.jsp) with FDR < 0.05 and fold enrichment >  = 2.

## Supplementary Information

**Additional file 1: Supplementary figure 1**. Histograms depicting the distribution of reproductive traits in among accession in FCCV_2016 experiment. (A) Reproductive traits are flower number (FLN), fruit number per inflorescence (FRN) and percentage of fruit set (FRS), studied in 2016 in three temperature regimens (T1: 25°C day/20°C night; T2: 30°C day/25°C night; T3: 35°C day/30°C night). (B) Correlations of FLN, FRN and FRS between temperature regimes ** *p<*0.01 *p<0.05. **Supplementary figure 2**. Histograms depicting the distribution of reproductive traits in among accession in FCCV_2017 experiment. (A) Reproductive traits are flower number (FLN), fruit number per inflorescence (FRN) and percentage of fruit set (FRS), studied in 2016 in three temperature regimes (T1: 25°C day/20°C night; T2: 30°C day/25°C night; T3: 35°C day/30°C night). (B) Correlations of FLN, FRN and FRS between temperature regimens ** *p<*0.01 *p<0.05. **Supplementary figure 3**. Histograms depicting the distribution of reproductive traits in among accession in ENZA_2018 experiment. (A) Reproductive traits are flower number (FLN), fruit number per inflorescence (FRN) and percentage of fruit set (FRS), studied in 2016 in three temperature regimens (T1: 25°C day/20°C night; T2: 30°C day/25°C night; T3: 35°C day/30°C night). (B) Correlations of FLN, FRN and FRS between temperature regimens ** *p<*0.01 *p<0.05. **Supplementary figure 4**. Graphic representation of minimum mean square comparisons for FRS (percentage of fruit set) of each genotype from FCCV-2017 and ENZA-2018 experiments by a two-way ANOVA in the three temperature experiments (T1: 25 °C/20 °C, T2: 30 °C/25 °C and T3: 35 °C/30 °C). **Supplementary figure 5**: Biological process enrichment in differentially expressed genes in the modern cultivar heat sensitive LA2660 between T2 and T3 (A) and heat tolerant LA2661 between T2 and T3 (B). Only categories with significant enrichment at *p<*0.05 and n≥3 are shown. **Supplementary figure 6**: Biological process enrichment of differentially expressed genes between the heat sensitive LA2660 and the heat tolerant LA2661 at T3. Only categories with significant enrichment at *p<*0.05 and n≥3 are shown. **Supplementary figure 7**: Biological process enrichment of differentially expressed genes in the “de penjar/da serbo” heat sensitive TRBA0160 between T2 and T3 (A) and heat tolerant TRVI0040 between T2 and T3 (B). Only categories with significant enrichment at *p<*0.05 and n≥3 are shown. **Supplementary figure 8**: Biological process enrichment of differentially expressed genes between the heat sensitive TRBA0160 and the heat tolerant TRVI0040 at T3. Only categories with significant enrichment at *p<*0.05 and n≥3 are shown. **Supplementary figure 9**: (A) Biological process and (B) molecular function enrichment of differentially expressed genes between the heat tolerant genotypes TRVI0040 and LA2661 inT3. Categories shown are significant at *p<*0.05 and n≥3.

**Additional file 2**: **Supplementary Table 1**: Details of the accessions analysed in the different experiments carrried out in the current study. Species, group, subgroup, donor, and the experiments in which they were analyzed are listed. **Supplementary Table 2**: Adjusted values for the 235 accession for the traits flower number (FLN), fruit number (FRN) and fruit set percentage (FRS) recalculated using the adjusted factor for each block at three temperature regimen T1(25ºC/20ºC), T2(30ºC/25ºC) and T3 (35ºC/30ºC). **Supplementary Table 3**: Values for fruit set percentage (FRS) at T1 (25ºC/20ºC), T2(30ºC/25ºC) and T3(35ºC/30ºC) of the 76 genotypes with significantly higher  FRS than the sensitive controls at least one high temperature regimen. Temperature regimen indicates at what regime the tolerance was observed: T2 (29 genotypes), T3 (25 genotypes) or at both temperature regimens T2_T3 (21 genotypes). **Supplementary Table 4**: Dunnett´s test to compare 16 putative heat tolerant genotypes and the control ‘MoneyMaker’ for flower number (FLN) and fruit number (FRN) in each of the four truss evaluated in MVCRI_2018 experiment.

## Data Availability

The datasets generated and/or analyzed during the current study are available in the Sequence Read Archive (SRA) repository under the accession number PRJNA678620, (https://www.ncbi.nlm.nih.gov/bioproject/PRJNA678620). A total of 32 sequences of ovary and fruit samples from LA2660, LA2661, TRVI0040 and TRBA160 in two temperatures (T2 and T3) were uploaded into the repository. All data generated or analyzed during this study are included in this published article and its supplementary tables and figures.
