# Supplementary material for: Identification of tomato accessions as source of new genes for improving heat tolerance: from controlled experiments to field
Source: BMC Plant Biol. 2021 Jul 22;21:345. doi: 10.1186/s12870-021-03104-4 (PMC8296629; doi:10.1186/s12870-021-03104-4)
Supplement: Supplementary file 1 — Additional file 1: Supplementary figure 1. Histograms depicting the distribution of reproductive traits in among accession in FCCV_2016 experiment. (A) Reproductive traits are flower number (FLN), fruit number per inflorescence (FRN) and percentage of fruit set (FRS), studied in 2016 in three temperature regimens (T1: 25°C day/20°C night; T2: 30°C day/25°C night; T3: 35°C day/30°C night). (B) Correlations of FLN, FRN and FRS between temperature regimes ** p<0.01 *p<0.05. Supplementary figure 2. Histograms depicting the distribution of reproductive traits in among accession in FCCV_2017 experiment. (A) Reproductive traits are flower number (FLN), fruit number per inflorescence (FRN) and percentage of fruit set (FRS), studied in 2016 in three temperature regimes (T1: 25°C day/20°C night; T2: 30°C day/25°C night; T3: 35°C day/30°C night). (B) Correlations of FLN, FRN and FRS between temperature regimens ** p<0.01 *p<0.05. Supplementary figure 3. Histograms depicting the distribution of reproductive traits in among accession in ENZA_2018 experiment. (A) Reproductive traits are flower number (FLN), fruit number per inflorescence (FRN) and percentage of fruit set (FRS), studied in 2016 in three temperature regimens (T1: 25°C day/20°C night; T2: 30°C day/25°C night; T3: 35°C day/30°C night). (B) Correlations of FLN, FRN and FRS between temperature regimens ** p<0.01 *p<0.05. Supplementary figure 4. Graphic representation of minimum mean square comparisons for FRS (percentage of fruit set) of each genotype from FCCV-2017 and ENZA-2018 experiments by a two-way ANOVA in the three temperature experiments (T1: 25 °C/20 °C, T2: 30 °C/25 °C and T3: 35 °C/30 °C). Supplementary figure 5: Biological process enrichment in differentially expressed genes in the modern cultivar heat sensitive LA2660 between T2 and T3 (A) and heat tolerant LA2661 between T2 and T3 (B). Only categories with significant enrichment at p<0.05 and n≥3 are shown. Supplementary figure 6: Biological process enri [file 12870_2021_3104_MOESM1_ESM.pptx]

## Slide 1
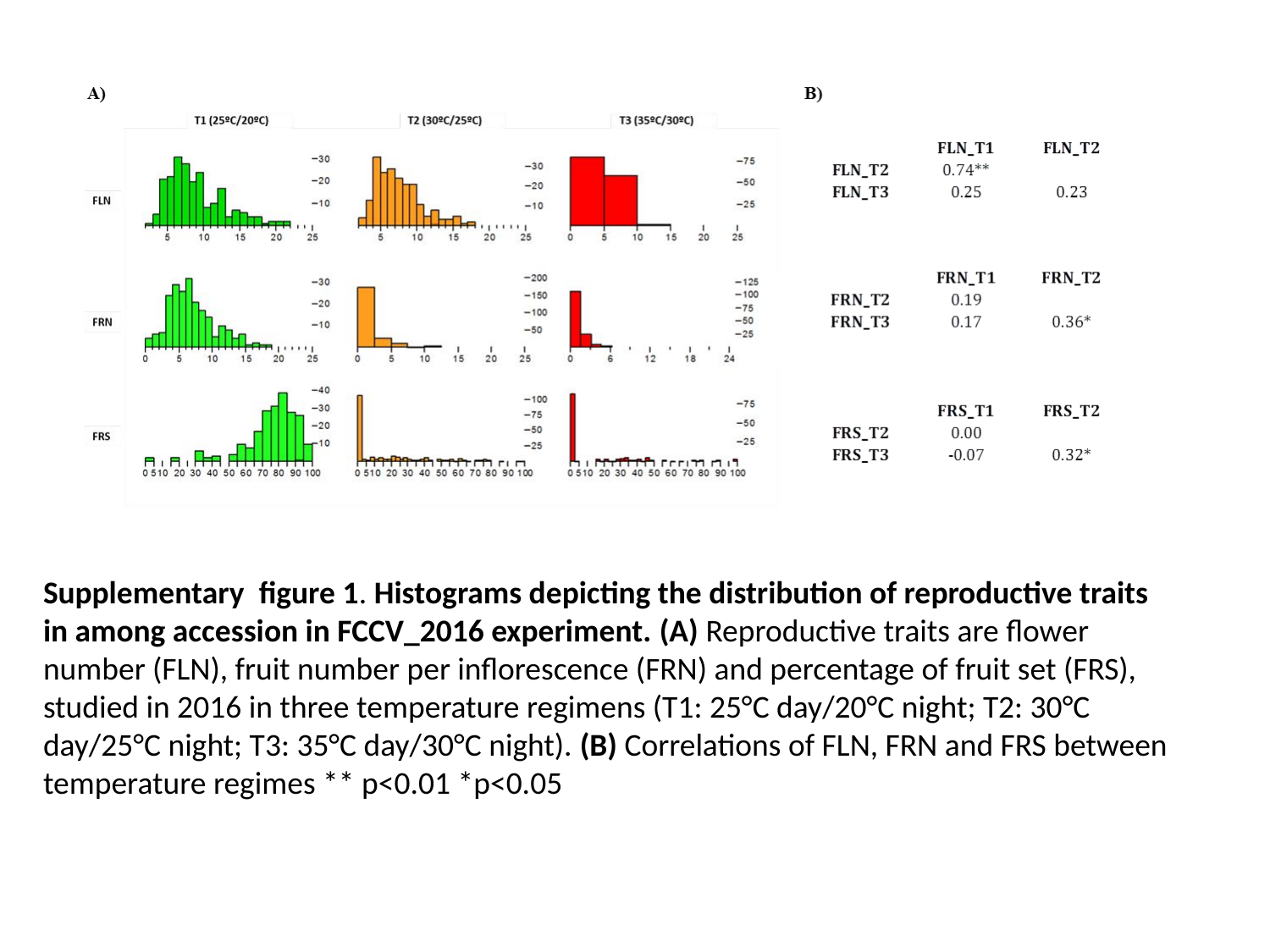

Supplementary figure 1. Histograms depicting the distribution of reproductive traits in among accession in FCCV_2016 experiment. (A) Reproductive traits are flower number (FLN), fruit number per inflorescence (FRN) and percentage of fruit set (FRS), studied in 2016 in three temperature regimens (T1: 25°C day/20°C night; T2: 30°C day/25°C night; T3: 35°C day/30°C night). (B) Correlations of FLN, FRN and FRS between temperature regimes ** p<0.01 *p<0.05

## Slide 2
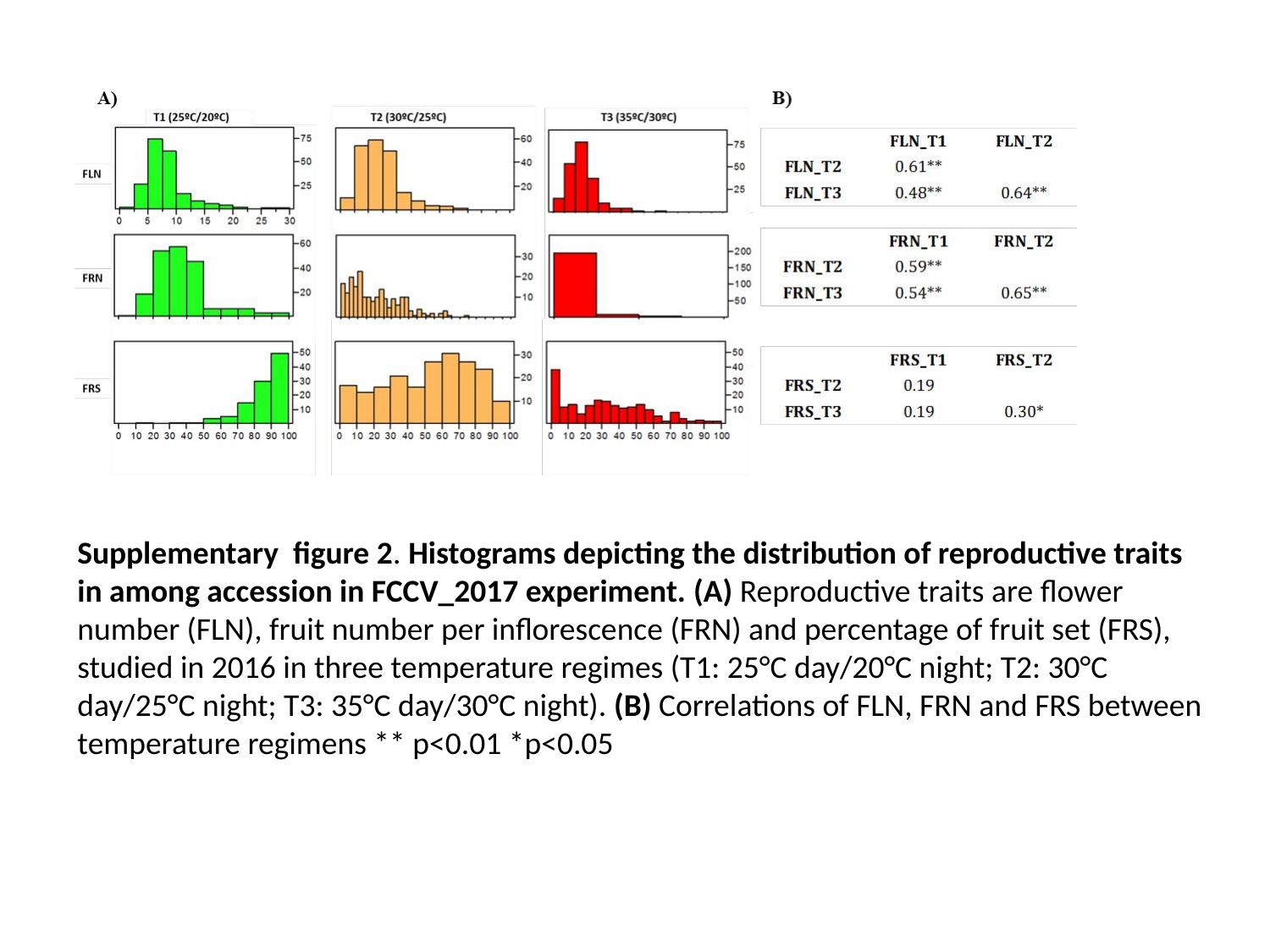

Supplementary figure 2. Histograms depicting the distribution of reproductive traits in among accession in FCCV_2017 experiment. (A) Reproductive traits are flower number (FLN), fruit number per inflorescence (FRN) and percentage of fruit set (FRS), studied in 2016 in three temperature regimes (T1: 25°C day/20°C night; T2: 30°C day/25°C night; T3: 35°C day/30°C night). (B) Correlations of FLN, FRN and FRS between temperature regimens ** p<0.01 *p<0.05

## Slide 3
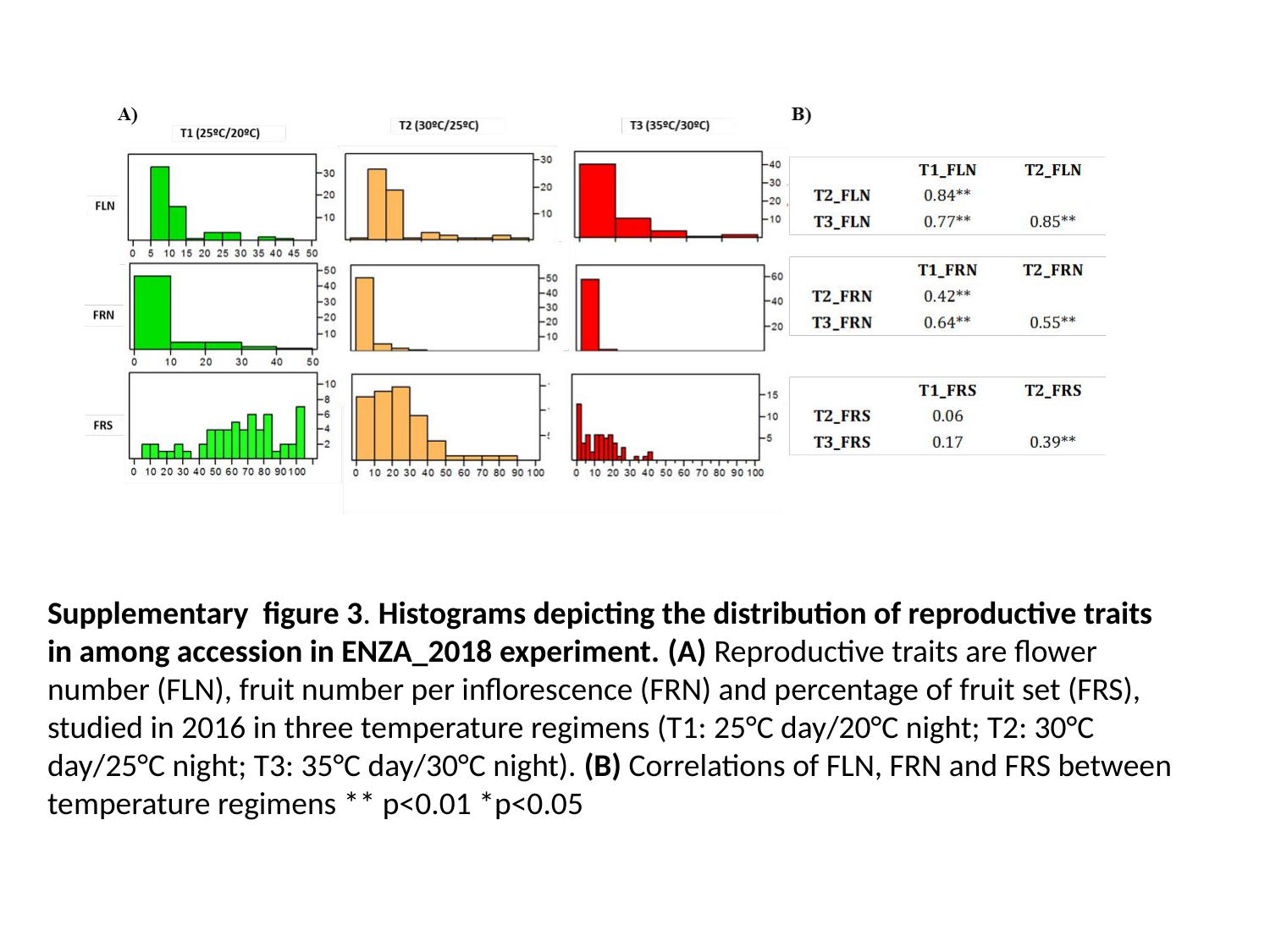

Supplementary figure 3. Histograms depicting the distribution of reproductive traits in among accession in ENZA_2018 experiment. (A) Reproductive traits are flower number (FLN), fruit number per inflorescence (FRN) and percentage of fruit set (FRS), studied in 2016 in three temperature regimens (T1: 25°C day/20°C night; T2: 30°C day/25°C night; T3: 35°C day/30°C night). (B) Correlations of FLN, FRN and FRS between temperature regimens ** p<0.01 *p<0.05

## Slide 4
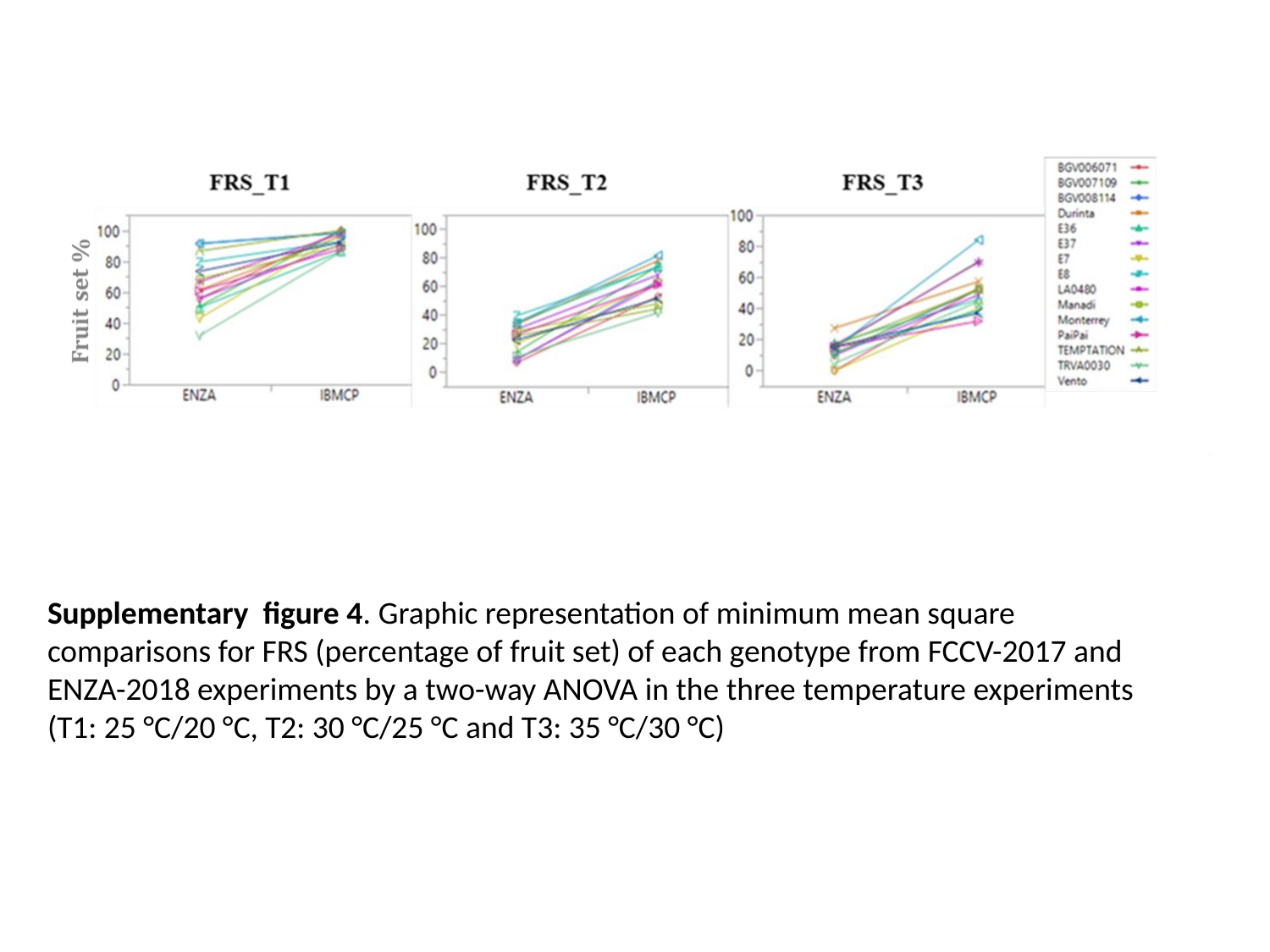

Supplementary figure 4. Graphic representation of minimum mean square comparisons for FRS (percentage of fruit set) of each genotype from FCCV-2017 and ENZA-2018 experiments by a two-way ANOVA in the three temperature experiments (T1: 25 °C/20 °C, T2: 30 °C/25 °C and T3: 35 °C/30 °C)

## Slide 5
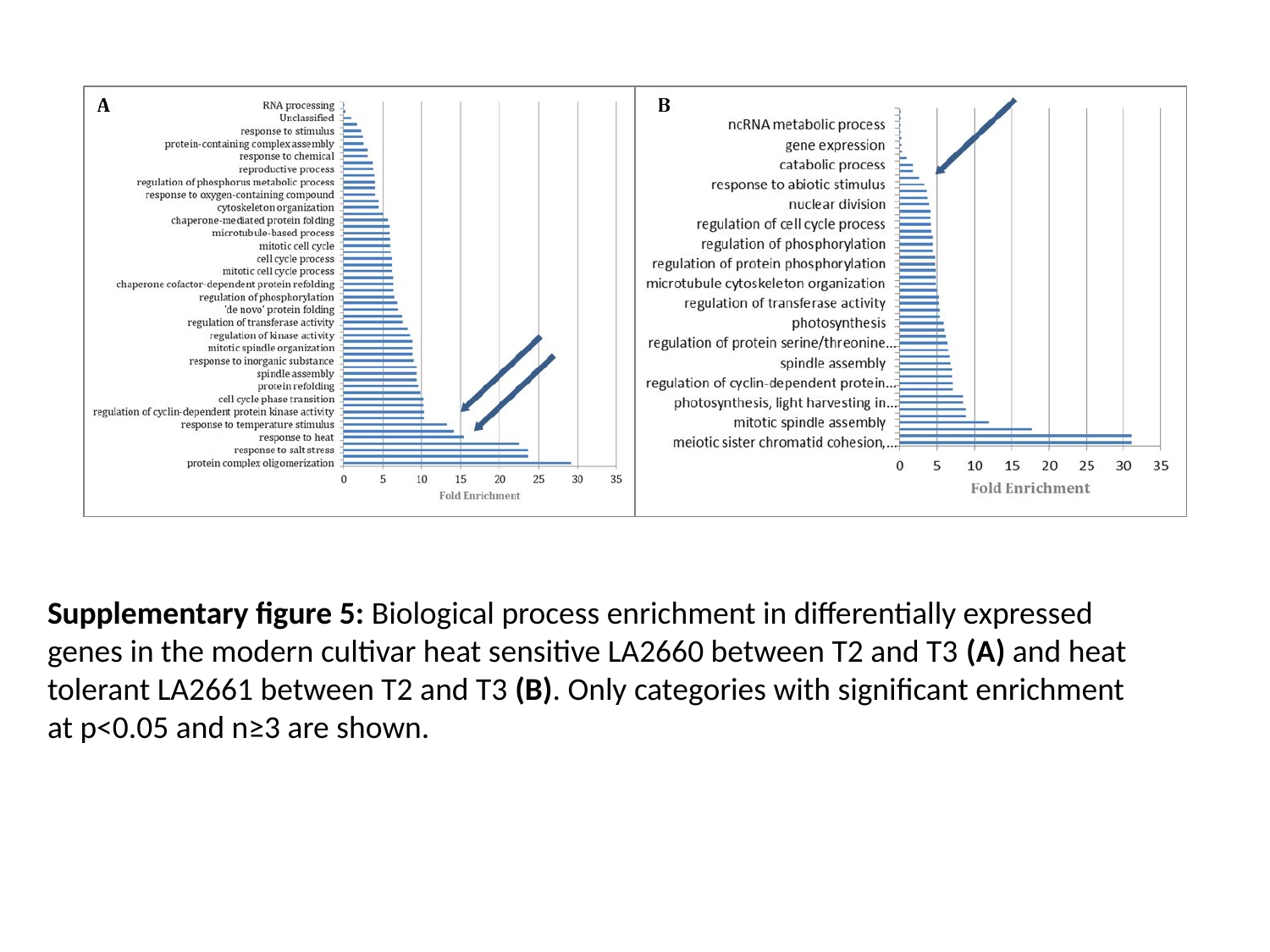

Supplementary figure 5: Biological process enrichment in differentially expressed genes in the modern cultivar heat sensitive LA2660 between T2 and T3 (A) and heat tolerant LA2661 between T2 and T3 (B). Only categories with significant enrichment at p<0.05 and n≥3 are shown.

## Slide 6
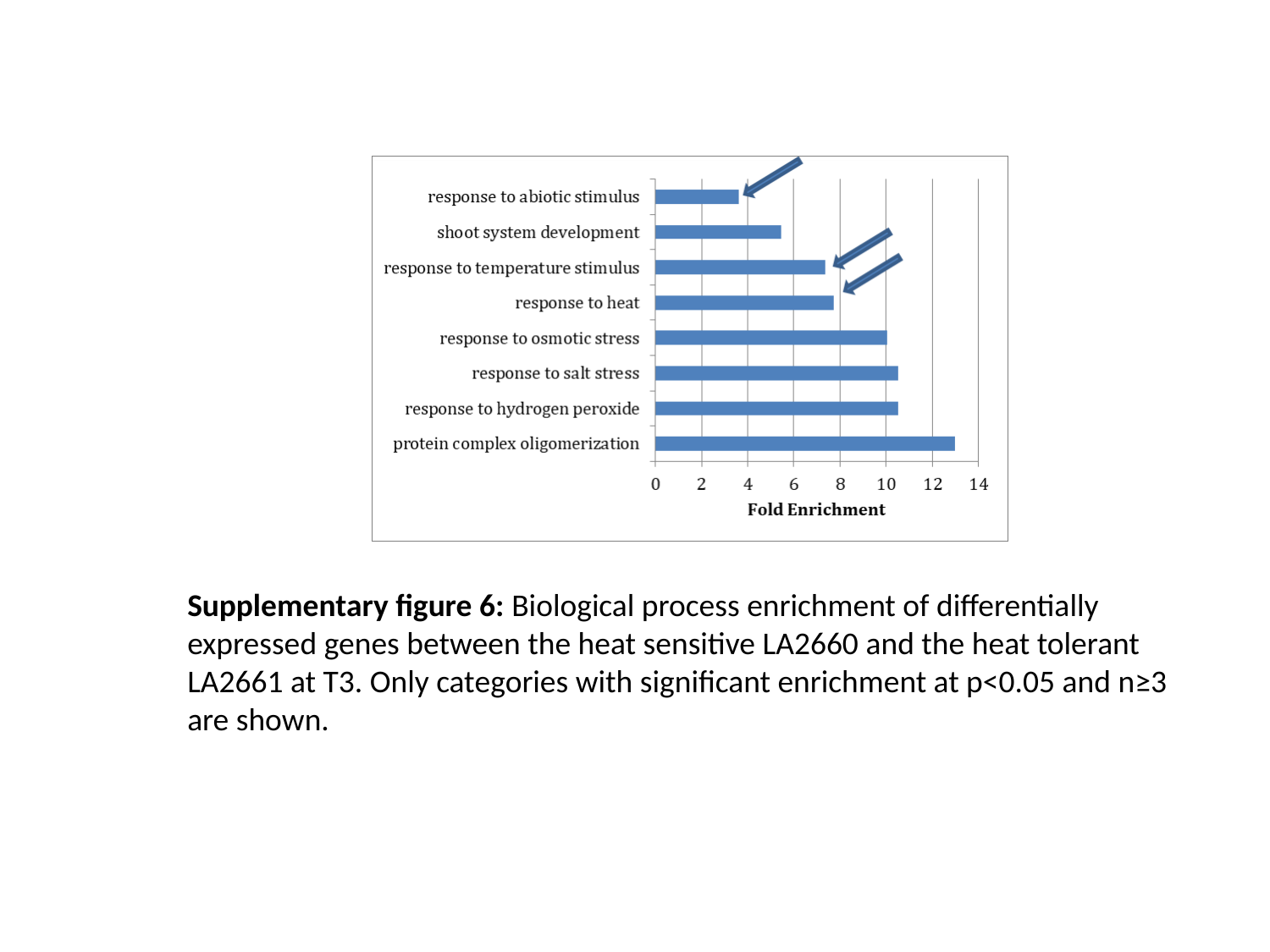

Supplementary figure 6: Biological process enrichment of differentially expressed genes between the heat sensitive LA2660 and the heat tolerant LA2661 at T3. Only categories with significant enrichment at p<0.05 and n≥3 are shown.

## Slide 7
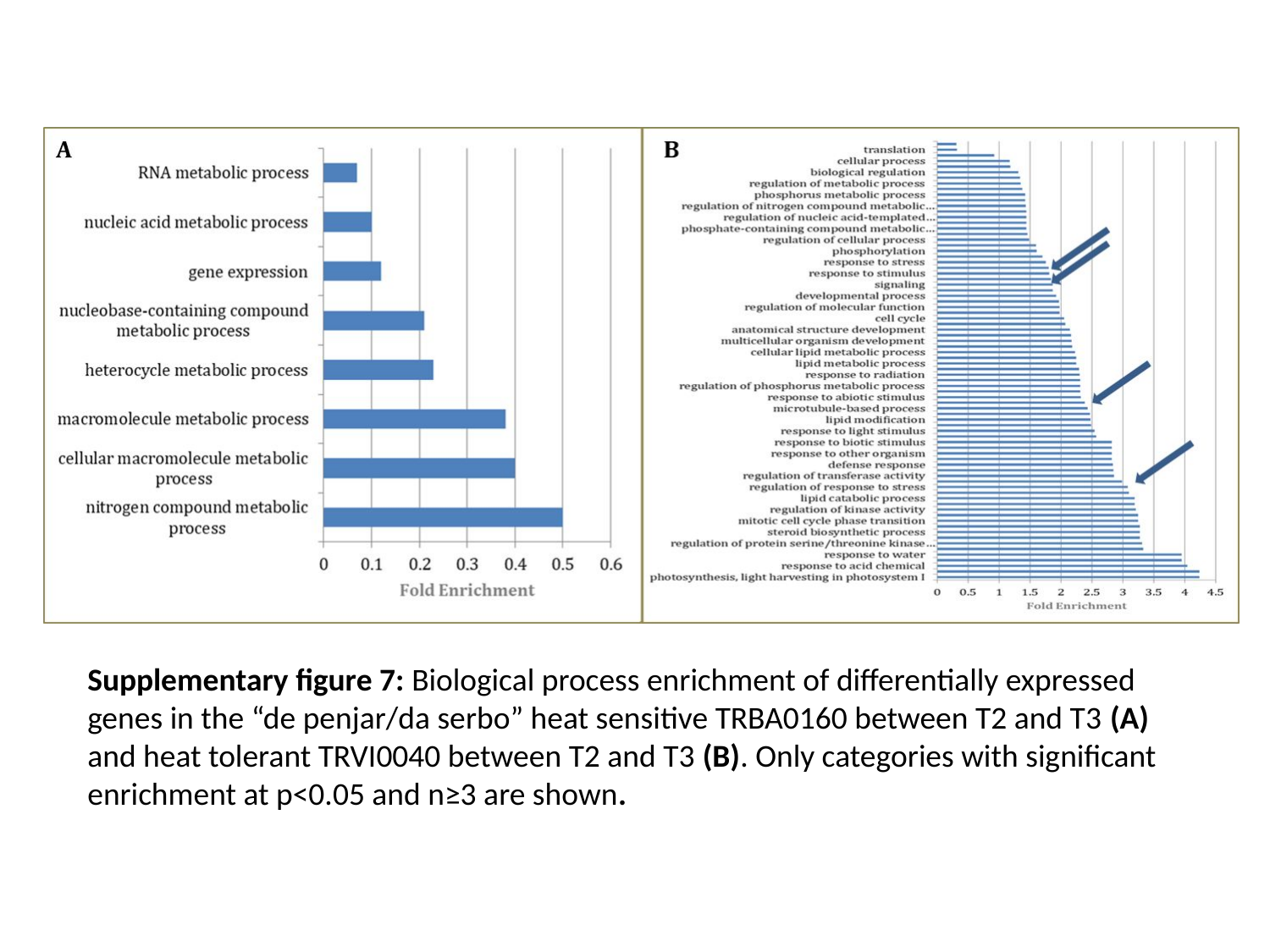

Supplementary figure 7: Biological process enrichment of differentially expressed genes in the “de penjar/da serbo” heat sensitive TRBA0160 between T2 and T3 (A) and heat tolerant TRVI0040 between T2 and T3 (B). Only categories with significant enrichment at p<0.05 and n≥3 are shown.

## Slide 8
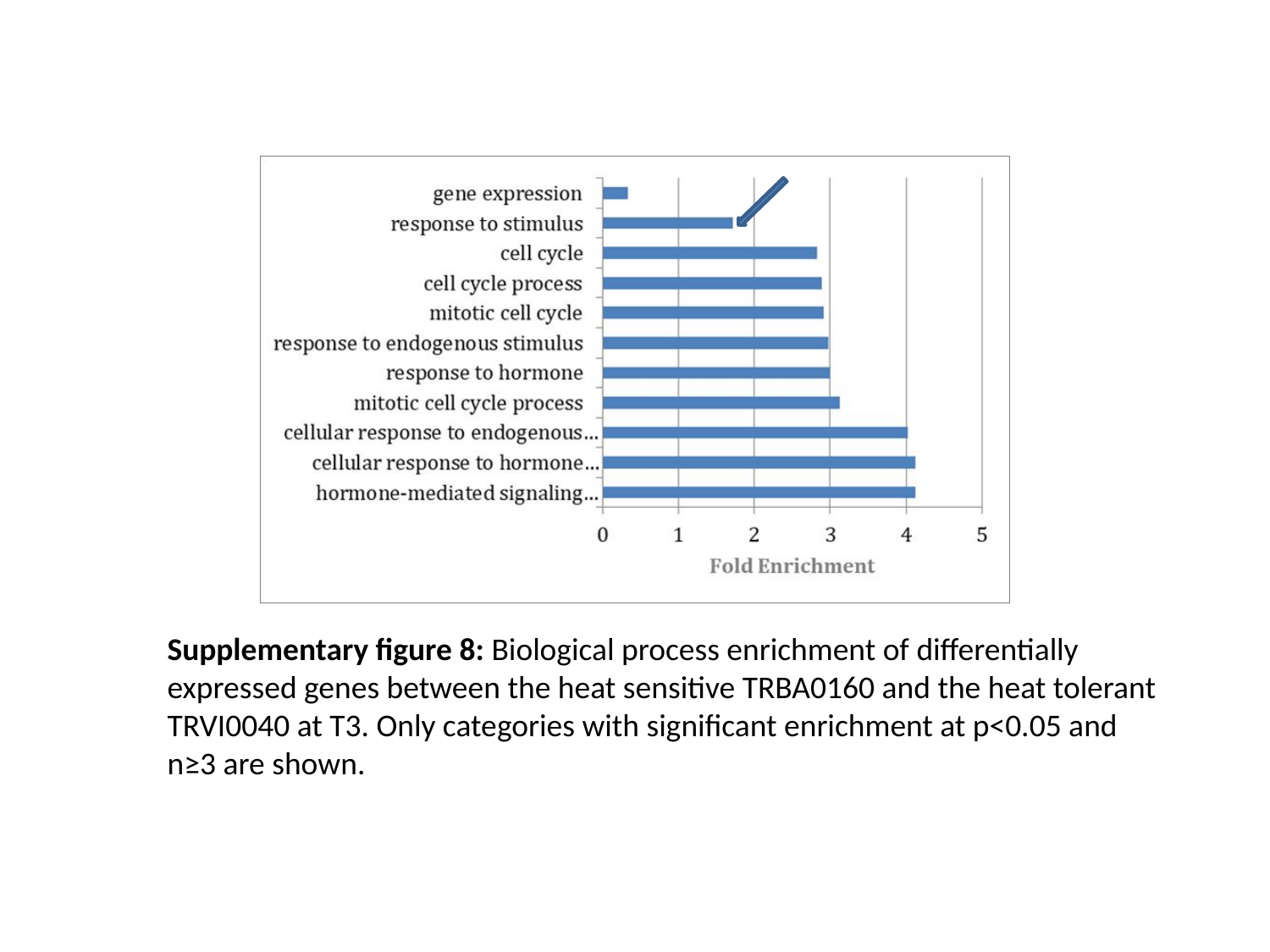

Supplementary figure 8: Biological process enrichment of differentially expressed genes between the heat sensitive TRBA0160 and the heat tolerant TRVI0040 at T3. Only categories with significant enrichment at p<0.05 and n≥3 are shown.

## Slide 9
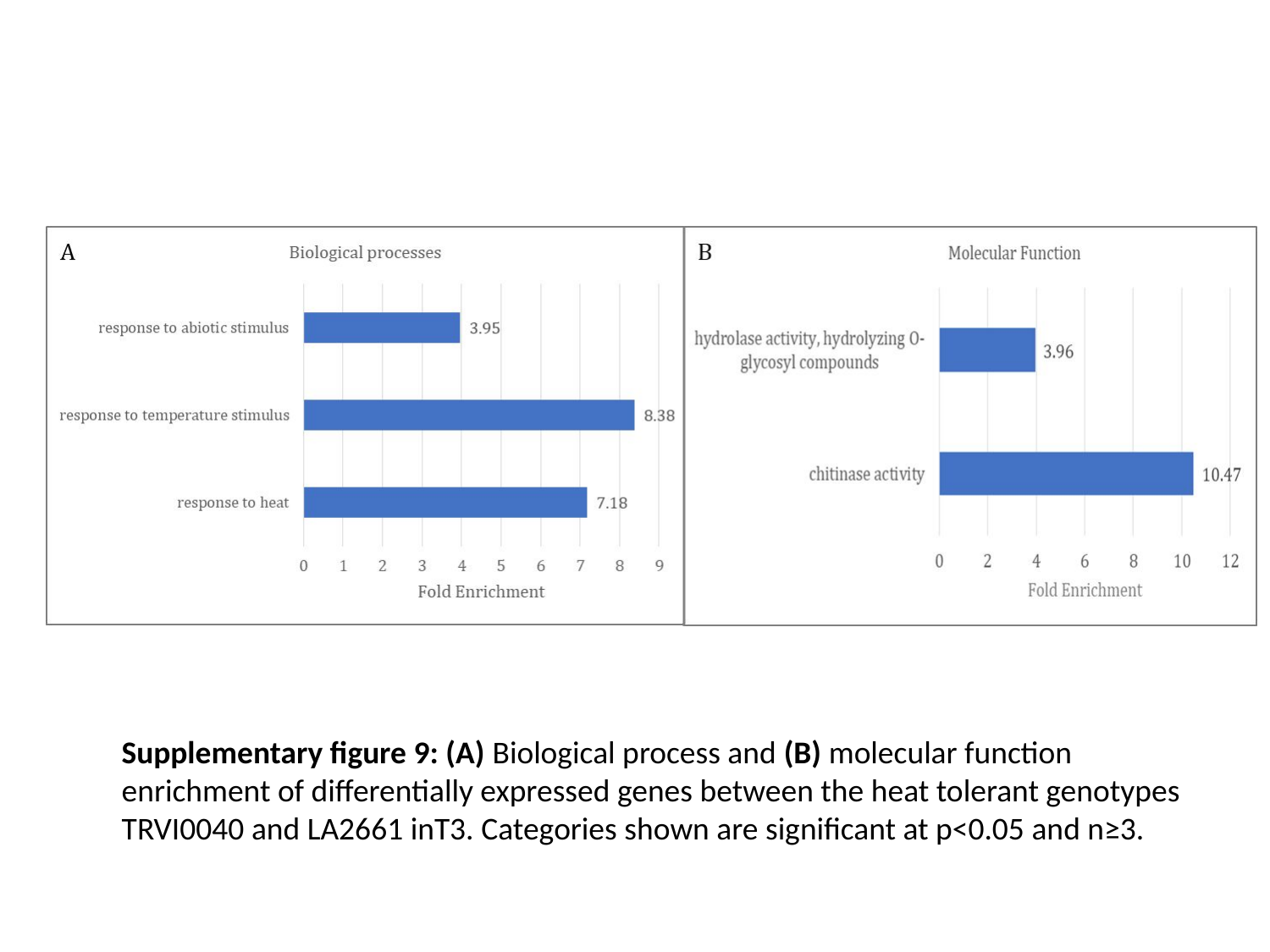

Supplementary figure 9: (A) Biological process and (B) molecular function enrichment of differentially expressed genes between the heat tolerant genotypes TRVI0040 and LA2661 inT3. Categories shown are significant at p<0.05 and n≥3.
